# Angiopoietin-2-induced blood–brain barrier compromise and increased stroke size are rescued by VE-PTP-dependent restoration of Tie2 signaling

**DOI:** 10.1007/s00401-016-1551-3

**Published:** 2016-03-01

**Authors:** Stefanie Gurnik, Kavi Devraj, Jadranka Macas, Maiko Yamaji, Julia Starke, Alexander Scholz, Kathleen Sommer, Mariangela Di Tacchio, Rajkumar Vutukuri, Heike Beck, Michel Mittelbronn, Christian Foerch, Waltraud Pfeilschifter, Stefan Liebner, Kevin G. Peters, Karl H. Plate, Yvonne Reiss

**Affiliations:** Institute of Neurology (Edinger Institute), Goethe University Medical School, Frankfurt, Germany; Department of Neurology, Goethe University Medical School, Frankfurt, Germany; Walter Brendel Centre of Experimental Medicine, Munich Heart Alliance, Ludwig-Maximilians-University, Munich, Germany; Deutsches Zentrum für Herz-Kreislauf-Forschung (DZHK RheinMain), Frankfurt, Germany; Aerpio Therapeutics, Cincinnati, OH USA

**Keywords:** Blood–brain barrier (BBB), Angiopoietin-2, Tie2 signaling, Stroke, Endothelium, Permeability, Vascular endothelial protein tyrosine phosphatase (VE-PTP)

## Abstract

The homeostasis of the central nervous system is maintained by the blood–brain barrier (BBB). Angiopoietins (Ang-1/Ang-2) act as antagonizing molecules to regulate angiogenesis, vascular stability, vascular permeability and lymphatic integrity. However, the precise role of angiopoietin/Tie2 signaling at the BBB remains unclear. We investigated the influence of Ang-2 on BBB permeability in wild-type and gain-of-function (GOF) mice and demonstrated an increase in permeability by Ang-2, both in vitro and in vivo. Expression analysis of brain endothelial cells from Ang-2 GOF mice showed a downregulation of tight/adherens junction molecules and increased caveolin-1, a vesicular permeability-related molecule. Immunohistochemistry revealed reduced pericyte coverage in Ang-2 GOF mice that was supported by electron microscopy analyses, which demonstrated defective intra-endothelial junctions with increased vesicles and decreased/disrupted glycocalyx. These results demonstrate that Ang-2 mediates permeability via paracellular and transcellular routes. In patients suffering from stroke, a cerebrovascular disorder associated with BBB disruption, Ang-2 levels were upregulated. In mice, Ang-2 GOF resulted in increased infarct sizes and vessel permeability upon experimental stroke, implicating a role of Ang-2 in stroke pathophysiology. Increased permeability and stroke size were rescued by activation of Tie2 signaling using a vascular endothelial protein tyrosine phosphatase inhibitor and were independent of VE-cadherin phosphorylation. We thus identified Ang-2 as an endothelial cell-derived regulator of BBB permeability. We postulate that novel therapeutics targeting Tie2 signaling could be of potential use for opening the BBB for increased CNS drug delivery or tighten it in neurological disorders associated with cerebrovascular leakage and brain edema.

**Electronic supplementary material:**

The online version of this article (doi:10.1007/s00401-016-1551-3) contains supplementary material, which is available to authorized users.

## Introduction

The blood–brain barrier (BBB) is a physiological barrier protecting the brain from circulating toxins and pathogens, yet providing essential substances such as iron and glucose. The BBB is composed of microvascular endothelium supported by astroglia and pericytes together forming the neurovascular unit (NVU) [1, 17]. BBB maintains brain homeostasis by tightly controlling the access of plasma components via intercellular tight junctions, efflux transporters expressed on its luminal membrane and a negligible rate of vesicular transcytosis. The strict regulation of the BBB is essential in proper CNS functioning [25]. The BBB is deregulated in a large variety of neurological disorders that include brain ischemia, traumatic brain injury, high-altitude cerebral edema, hepatic encephalopathy and many types of brain tumors. BBB deregulation can lead to brain edema and increased intracranial pressure, further deteriorating the clinical outcome. Thus, there is an urgent clinical need for novel strategies to prevent or treat BBB deregulation [45, 56].

Angiopoietin-1 (Ang-1) and angiopoietin-2 (Ang-2) are secreted growth factors that exert downstream signaling through the Tie2 receptor tyrosine kinase [5, 24, 47]. Tie2 is predominantly expressed in endothelial cells and controls vessel remodeling and maturation [23, 47, 51]. Ang-1 activates the Tie2 receptor by phosphorylation, while Ang-2 is able to prevent receptor phosphorylation by competitive binding in a context-dependent manner, thus leading to inactivation of Tie2 and facilitating angiogenesis [5, 19, 28, 39, 47, 58]. Accordingly, Ang-2 is not expressed in the normal adult brain endothelium, but upregulated in brain tumors such as glioblastoma [32, 52, 57, 63]. Ang-1, in contrast, has shown to be constitutively expressed in non-endothelial cells, such as pericytes [47]. These findings suggest that Tie2 signaling in activated endothelium is mainly regulated via the expression of Ang-2 [47, 52]. We have previously demonstrated that Ang-2 overexpression in endothelial cells leads to an increase in myeloid cell infiltration via the β_2_-integrins [53]. This finding was supported by data obtained from human biopsies of inflamed tissues dominated by myeloid cell infiltration, where Ang-2 was upregulated in endothelial cells [53]. Other studies indicated increased vascular permeability in peripheral endothelial cells upon Ang-2 expression that support the increased infiltration we observed [11, 64]. Furthermore, we found the highest levels of Ang-2 in myeloid-dominated inflamed brain tissues [53], prompting us to study the role of Ang-2 in cerebrovascular permeability that is tightly regulated by the BBB.

Ang-1 has been shown to result in vessel stabilization and recruitment of mural cells, whereas Ang-2 leads to vessel destabilization and pericyte dropout [5, 24, 47]. Pericytes have been demonstrated to regulate neurovascular permeability [4, 10, 18], suggesting a role for Angiopoietins at the BBB. Of note, Ang-1 has been reported to prevent vascular leakage [59, 60], while Ang-2 has been shown to increase vascular permeability in non-brain endothelial cells in vitro and in vivo [11, 64]. However, the precise role of Ang-2 at the BBB remains unclear.

To investigate the influence of Ang-2 at the BBB, we used primary mouse brain microvascular endothelial cells (MBMECs) for in vitro permeability measurements and performed in vivo studies with Ang-2 GOF mice which express Ang-2 in an inducible, endothelial-specific manner (Ang-2 Tet-OS: Tie1 tTA) that we employed previously [46, 52, 53]. Expression analyses were carried out on freshly isolated brain microvessels (MBMVs), followed by localization and ultrastructural analyses in vivo (by means of immunofluorescence and electron microscopy). We have previously demonstrated increased Ang-2 levels in an experimental ischemia model in rodents [7, 8]. Here, we extend these observations by employing permeability studies using Ang-2 gain-of-function mice [46]. Permanent and transient mouse models of middle cerebral artery occlusion were employed to this end. Therapeutic rescue was assessed at 24 h post-stroke, a time point characterized by marked BBB damage using a VE-PTP inhibitor that activates Tie2 signaling.

## Materials and methods

### Animals

The present study was performed in accordance with the German Protection of Animals Act and the guidelines for care and use of laboratory animals by the local committee (Regierungspraesidium Darmstadt, approval number FK/1044). Mouse lines comprising a Tie1 tTA driver and a TetOS human Ang-2 responder transgene were generated as described previously [46]. Mice received doxycycline containing food pellets (100 mg/kg, ssniff Spezialdiaeten GmbH, Soest, Germany) during breeding. Transgene overexpression was induced from birth (P0) in newborn littermates by withdrawing doxycycline. Upon doxycycline removal mice start displaying elevated serum levels of human Ang-2 [46]. Genotypes were determined by PCR analysis and expression levels of hAng-2 were monitored by ELISA. Unless otherwise noted, all the mice employed were adult (8–12 weeks, 25–30 g body weight) CD1 strain without gender discrimination. For transgenic mice, controls were always littermates. Details about animal experiments are presented in Suppl. Table 6.

### Ethics statement for human specimens

All studies on human subjects were covered by an ethics statement. The ethics approval number for autopsy material is GS-249/11 and for resection material GS-04/09.

### Statistical analysis

All statistical analyses were performed using Prism 5.0 software (GraphPad). A value of *p* < 0.05 was considered to be statistically significant. Data in graphs are represented as mean ± SEM with *p < 0.05; **p < 0.01; ***p < 0.001. The number of animals and experimental repeats are presented in Suppl. Table 6. This information is also indicated in the corresponding figure legends and the specific statistical test employed is indicated in the corresponding methods section.

### ELISA studies

We monitored hAng-2 levels by ELISA in Ang-2 GOF mice as previously described [46]. In addition, hAng-2 serum levels were determined in healthy human volunteers and stroke patients using the hAng-2 Quantikine ELISA kit according to the manufacturer’s instructions (R&D Systems). Detailed patient data are provided in the Suppl. Table 4 (healthy volunteers, *n* = 16; lacunar stroke patients, *n* = 4; territorial stroke patients, *n* = 15). Statistical analysis was performed by one-way ANOVA followed by Dunnett’s post hoc test.

### Permanent middle cerebral artery occlusion (pMCAO)

Experiments were performed as described previously [9]. In detail, animals were anesthetized intraperitoneally (i.p.) with a combination of midazolam/medetomidine/fentanyl (5/0.5/0.05 mg/kg body weight). Using an operating microscope, the skin between the lateral part of the left orbit and the left external auditory meatus was incised. The top and back segments of the temporal muscle were transected, and the skull was exposed by retraction of the muscle. A small opening (1–2 mm in diameter) was made with a handheld drill in the frontal bone about 1 mm rostral to the fusion of the zygoma and squamosal bone, and 3.5 mm ventral to the dorsal surface of the brain. The MCA was exposed after the dura was opened and retracted. The MCA was exposed and occluded by ligation using 10-0 nylon thread (Ethicon) and transected distal to the point of ligation. The retracted soft tissues were replaced, the wounds were sutured and the mice were placed back into their cages. Anesthesia was terminated by i.p. injection of atipamezole/naloxone/flumazenil 2.5/1.2/0.5 mg/kg body weight).

Following pMCAO, WT and Ang-2 GOF mice (C57BL/6 background) were sacrificed at 24, 72 h and 7 days. Brains were isolated, embedded in Tissue TEK^®^ O.C.T. and stored at −80 °C until use. Brain sections on objective slides were dried for 15 min at 37 °C, followed by dehydration in 80 % ethanol for 2 min and drying for 5 min. The samples were then washed 3× in ddH_2_O and incubated in Giemsa staining solution (Merck) for 1 h at RT. The sections were immersed in glacial acetic acid for a few minutes and thereupon incubated in 96 and 100 % ethanol and xylene. Sections were mounted with Entellan (Merck). Images of infarct brain sections were taken and analyzed with a Stereo Investigator (MicroBrightField, Inc.). The infarct areas were measured for serial sections (spaced 100–200 µm) and these were combined to obtain the volume of the infarct size in mm^3^ taking into account the section thickness for volume calculation. The statistical analysis of stroke sizes was performed by two-tailed unpaired *t* test. Details about animal numbers are included in Suppl. Table 6.

### Transient MCAO (tMCAO)

The operator was blinded to the genotype and treatment of mice. AKB-9785 was pre-administered at 14 and 2 h prior to surgery and 10 h post-surgery following tMCAO. CD1 male mice were injected subcutaneously with AKB9785 (30 mg/kg) or vehicle (dH_2_O) as a control. MCAO surgeries were performed as described previously [44]. Briefly, mice were anesthetized with 1.5 % isofluorane followed by right MCA occlusion using standardized monofilament (0.23 mm tip diameter, Doccol). 30 min after the occlusion, the filament was removed and 24 or 72 h post-occlusion mice were subjected to neurological deficit scoring according to 14-point modified neurological severity scores (mNSS) [14]. Mice were then killed and the brain was isolated. The cerebellum and olfactory lobes were removed and subsequently the brain was cut into 2 mm-thick slices on a brain matrix. For each animal, three brain slices were transferred to 2,3,5-triphenyltetrazolium chloride (TTC, 2 %). One additional slice was either utilized for native embedding into O.C.T. compound or stored in RIPA buffer plus proteinase/phosphatase inhibitors for Western blot analysis. The stroke sizes were determined and analyzed by two-tailed unpaired *t* test. Stroke incidence was in the range of 80–90 % and animals were only excluded when there was no stroke based on TTC staining. More than 80 % of animals survived MCAO surgery. The details are included in Suppl. Table 6.

### In vivo tracer studies

Tracer experiments were performed in healthy WT or GOF mice and not in stroke-induced animals unless otherwise specified. A mixture (1:2) of Texas Red (TXR) or tetramethylrhodamine (TMR) 3 kD dextran (1 mM, Invitrogen) and Lucifer Yellow (LY; 10 mM, Sigma) was injected into the tail vein of mice (100 µl/mouse) and allowed to circulate for 4 min. This step was followed by anesthesia (as described before) and transcardial perfusion for 1 min with PBS. Brain and kidneys were isolated followed by homogenizing hemi-brain or a single kidney in PBS at 4 °C, and the remaining kidney and hemi-brain were frozen down in Tissue TEK^®^ O.C.T. Compound (Sakura) on dry ice for immunohistochemical analysis. After spinning down the samples at 10,000×*g* for 15 min at 4 °C, the supernatant was measured to obtain raw fluorescence units (RFU) in a fluorescence plate reader (Tecan) at excitation/emission wavelength of 425/525 nm for LY and 595/625 nm for TXR-3 kD dextran or 550 nm/580 nm for TMR 3 kD dextran and normalized to the serum RFUs from blood withdrawn immediately before perfusion. Kidneys were used as a positive control for tracer injections. In experiments using larger molecular weight tracers, 2 % Evans blue dye (70 kD, albumin-bound form) was injected intravenously into the tail vein of mice and circulated for 2 h. After transcardial perfusion with PBS, isolated brains were minced with a scalpel and transferred into a tube containing formamide. Samples were incubated at 67 °C for 24 h followed by centrifugation at 10,000×*g* for 1 h at 4 °C. The absorbance of the supernatants was measured at 620 nm in a plate reader (Tecan). Statistical analysis of in vivo tracer experiments was performed by two-tailed unpaired *t* test.

### Isolation of mouse brain microvessels (MBMVs)

Brains were isolated and rolled on a Whatman filter membrane (Schleicher & Schuell) to peel off meninges. They were subsequently pooled, homogenized in MVB buffer [26] using a dounce homogenizer (Wheaton, 0.025 mm clearance) and centrifuged at 400×*g* for 10 min at 4 °C. The pellet was resuspended in 25 % BSA and centrifuged at 1500×*g* for 30 min to remove myelin fat that was aspirated carefully from the top layer. The microvessel pellet containing red cells and nuclei was resuspended in MVB buffer and filtered through a 40 µm nylon mesh (BD). For protein analysis, microvessels were resuspended in HES buffer [21] with protease and phosphatase inhibitor cocktails (Roche) and stored at −80 °C. For mRNA expression analysis, the final microvessel sample was lysed in RLTplus buffer (Qiagen) and stored at −80 °C until use.

### Isolation of mouse brain microvascular endothelial cells (MBMECs)

A modified protocol was utilized to isolate endothelial cells for in vitro cell culture experiments [37, 43]. Briefly, meninges-free brains were pooled and homogenized as described above followed by digesting the pellet with 0.75 % collagenase II (Worthington) in buffer A (1:1:1 volume ratio) for 1 h with shaking at 37 °C. For the removal of myelin, samples were resuspended in 25 % BSA and centrifuged at 1500×*g* for 30 min at 4 °C followed by enzymatic digestion of the pellet with Collagenase/Dispase (Roche) and DNase I (Worthington) in buffer A for 12 min at 37 °C. After centrifugation, MBMECs were resuspended in MCDB-131 complete medium [15] and seeded on six-well plates pre-coated with type 1 collagen (150 µg/cm^2^, Corning). After 4 h, the medium was changed to puromycin (5 µg/ml) containing medium, which selects for ECs as they possess p-glycoprotein (P-gp) that effluxes out puromycin, a P-gp substrate [22, 37]. Contaminating cells do not possess P-gp and hence do not survive the treatment. The medium was changed back to puromycin-free medium after 3 days.

### Culture of bEnd5 cells

To reduce the number of animals for MBMECs, immortalized brain endothelial cells (bEnd5) were obtained and cultured as described previously in MCDB-131 complete medium [15]. Dishes were gelatin coated (0.1 %, 30 min), followed by seeding the cells at 50,000 cells/cm^2^. For Western blotting experiments, cells were cultured for 5–6 days to obtain a confluent monolayer followed by treatments in serum-free medium.

### Culture and treatment of murine astrocytes and human pericytes

Astrocytes were isolated and cultured from postnatal mouse brains (0–4 days) as described previously [61]. They were then seeded onto 24-well plates pre-coated with 0.001 % poly-l-lysine for 30 min. Primary human brain vascular pericytes (ScienCell) were also seeded in a similar way. Upon confluency, the cells were treated with 0.1 % BSA/PBS for 16 h which served as control, or with 500 ng/ml recombinant human Ang-2 for 16 h plus/minus 1 µM AKB-9785 for 10 min followed by washing with PBS. Cells were lysed for 15 min on ice in RIPA buffer (Sigma) containing one tablet each of protease and phosphatase inhibitor cocktail (Roche). Following this, the samples were transferred into Eppendorf tubes and centrifuged at 16,000×*g* at 4 °C for 10 min. The supernatants were transferred into new Eppendorf tubes and stored at −80 °C until further use.

### Transendothelial electrical resistance (TEER)/capacitance (Ccl) measurements

MBMECs (100,000 cells/cm^2^, 4–6 mice/prep) isolated from CD1 wild-type mice were seeded onto 1 µm-pore 24-well PET transwell inserts (Greiner Bio-One) pre-coated with fibronectin (5 µg/cm^2^, Sigma-Aldrich). The inserts were transferred to a cellZscope^®^ device (NanoAnalytics) placed in a humidified incubator (37 °C, 5 % CO_2_) and TEER and Ccl values were obtained from continuous impedance measurements as described previously [15]. Treatment with hAng-2 (500 ng/ml) with or without AKB-9785 (1 µM) was started upon reaching a plateau in TEER/Ccl levels and continued up to 48 h. The TEER/Ccl measurements were statistically analyzed by two-way ANOVA with matching to account for inter-prep variations.

### In vitro permeability assay

MBMECs were isolated from C57BL/6 mice pooling four to six animals in each preparation and cultured as described above on transwell inserts. Confluent cells were treated with recombinant hAng-2 (500 ng/ml) for 24 h. This was followed by performing the permeability assay for 0.45 kD LY (Sigma), TXR-3 kD dextran (Invitrogen), TMR-20 kD dextran (Sigma) and FITC-70 kD dextran (Sigma) as described previously [15]. Briefly, the tracer mix was added to the apical chamber and media aliquots were collected from the top and bottom chamber at 1 h time point. The samples were read in a fluorescence plate reader (Tecan) at the corresponding tracer excitation/emission and permeability obtained as bottom chamber fluorescence normalized to the apical chamber fluorescence with the ratio for the control condition set to 100 %. For filipin experiments, cells were treated with hAng-2 (500 ng/ml, 1 h) alone or with AKB-9785 (1 µM) followed by filipin treatment (10 nM, 30 min). Permeability assay was then performed as above and top/bottom aliquots collected after 1 h and read in a plate reader. The in vitro permeability assays were statistically analyzed by one-way ANOVA with matching to account for inter-prep variations with Tukey post hoc test for multiple comparisons.

### Immunohistochemical staining and quantification of murine cryosections

Brains were isolated and frozen in native state in Tissue TEK^®^ O.C.T. Compound (Sakura) on dry ice and stored at −80 °C. For all stainings, unless specified otherwise, frontal coronal sections (10 µm, at the starting point of ventricles) were fixed in 95 % ethanol for 5 min at 4 °C and by acetone fixation for 1 min at room temperature (RT) followed by incubation with primary antibodies (Table 1) for 1.5 h at RT. Samples were post-fixed in 4 % PFA and mounted with AquaPoly Mount (Polysciences). Images (5/section) were acquired with a C1si confocal microscope (Nikon) with 20× and 60× objectives. The number of desmin-positive cells were counted using NIS Elements AR Imaging Software 4.10 (Nikon) and expressed in percent normalized to number of CD31-positive cells. A two-tailed unpaired *t* test was performed for statistical significance. The astrocytic endfeet coverage was analyzed by the number of endfeet processes normalized to the number of CD31-positive vessels at 20× magnification. The length of the astrocytic endfeet processes was obtained by analyzing three images per animal at 60× magnification. A two-tailed unpaired *t* test was performed for statistical significance.

**Table 1:** Antibodies for immunohistochemical analysis

Antibody	Marker for	Company	Catalog #	Dilution
CD31	Endothelial cells	BD	550274	1:100
Desmin	Pericytes	Dako	M0760	1:200
Collagen type IV	Collagen type IV	Abd Serotec	2150-1470	1:250
Aquaporin-4	Astrocytes	Millipore	AB2218	1:200
Human Ang-2	Ang-2	R&D	MAB 0983	1:100
IBA1	Microglia	Wako	019-19741	1:1000
pTie2	Phosphorylated Tie2	R&D	AF2720	1:50
Tie2	Tie2	R&D	AF762	1:50

The same procedure was followed for IgG staining from either PFA-fixed (tMCAO) or native (pMCAO) samples. Three equal area regions of interest (ROI) around the vessels were chosen for the analysis of mean intensity from a single image per animal by NIS Elements AR Imaging Software 4.10 (Nikon). A two-tailed unpaired *t* test was employed for statistical analysis.

For pTie2/Tie2 staining, the frontal coronal sections were fixed immediately post-sectioning in ice-cold methanol/acetone for 10 min, air-dried and stored at −80 °C until use. Sections were washed once in PBS and blocked for 60 min in 20 % NDS/1 % BSA in PBS. The samples were incubated in primary and secondary antibodies (diluted in 1 % BSA/PBS) at RT for 1.5 h each step. Image acquisition was performed as mentioned above using 40× objectives (*n* = 5 per group were analyzed).

### Immunohistochemical staining and quantitation of murine vibratome sections

For quantitative analysis of pericyte coverage in brain vasculature, mice (*n* = 3 for each genotype) were anesthetized and transcardially perfused with PBS for 1 min as described earlier followed by 4 min with 4 % PFA. The brains were dissected and post-fixed overnight in 4 % paraformaldehyde. Serial 50 µm coronal sections were cut on a Leica Vibratome (VT 1000S). At least four sections at intervals of 500 µm were analyzed on a Nikon C1 Spectral Imaging Confocal Laser Scanning Microscope System, using NIS Elements Microscope Imaging Software (Nikon Instruments, Inc., Düsseldorf, Germany). Video clips were prepared using Imaris 7.6.5, Bitplane Scientific Software.

The following primary antibodies were used: mouse monoclonal anti-desmin (#M0760, Dako, 1:100) and rabbit polyclonal anti-collagen IV (#2150-1470, Abd serotec, 1:250). Antigen retrieval was performed prior to the staining procedure by heating the sections in a 10 mM citric acid buffer, pH 6.0. Samples were incubated with primary antibodies at 4 °C over night followed by incubating with the following secondary antibodies: goat anti-mouse IgG Alexa Fluor 488 and goat anti-rabbit IgG Alexa Fluor 647.

### Immunohistochemical staining and quantification of human stroke samples

Human brain sections derived from 28 autopsy specimens (Suppl. Table 5) pre-fixed in 10 % formaldehyde and paraffin embedded were first de-paraffinized followed by antigen retrieval. Hematoxylin/Eosin staining was performed according to standard protocols, followed by histopathological grading (stroke grades I–III) according to Mena et al. [40]. A Ventana Benchmark automated system was used for hAng-2 staining (Table 2) using standard protocol utilizing cell conditioning 1 (pre-diluted, Ventana Medical Systems) solution for antigen retrieval. Primary antibody signal was detected with IHC Ultra Map AT kit (Ventana Medical Systems). From the acquired images, Ang-2-positive vessels were counted and given a score from 0 to 3 (with 0 being no Ang-2 signal and 3 being the maximum; *n* = 13 cases, whiskers plot (2.5–97.5 percentile), Kruskal–Wallis test with Dunn’s multiple comparison test).

**Table 2:** Antibodies for Western blot analysis

Antibody	Company	Catalog #	Dilution
α-Tubulin	Sigma-Aldrich	T6199	1:500
Claudin-5	Invitrogen	35-2500	1:500
β-Catenin	BD	610154	1:500
Caveolin-1	Santa Cruz	SC-894	1:500
Glut-1	See Ref. [21]	Not applicable	1:1000
β-Actin	Santa Cruz	sc-69879	1:1000
pTie2	R&D	AF2720	1:400
Tie2	R&D	AF762	1:500
pVE-cadherin	Abcam	ab27776	1:1000
VE-cadherin	Santa Cruz	sc-6458	1:500
pAkt	Cell Signaling	4060S	1:1000
Akt	Cell Signaling	9272S	1:1000

### Western blotting

Samples were solubilized in urea/SDS buffer (2.3 M urea, 1.5 % SDS, 50 mM Tris, 25 mM TCEP and 0.01 % BPB) for 1.5 h at 30 °C with shaking [21]. The solubilized samples were subjected to electrophoresis on Tris–HCl bis-acrylamide gels (8–15 %) and subsequently transferred to nitrocellulose membranes at 4 °C ON. The membranes were blocked for 1 h at RT in 1x Roti^®^-block (Roth) and incubated with primary antibodies (Table 2) overnight at 4 °C, washed with PBS-T (0.5 % Tween-20) and incubated with horseradish peroxidase-conjugated or fluorescent secondary antibodies (LI-COR) for 1 h at RT. The membranes were imaged in Odyssey (LI-COR) imaging device and not by classical chemiluminescence using films and therefore produced very little background. An artificial black and white color has been included for the color of the blots and hence appears overly processed. All the quantification was however done on raw images using Image Studio 2.1 software (LI-COR) as described previously [21, 22]. The Western blots of MBMV were statistically analyzed by two-tailed unpaired *t* test (7 mice/prep). MBMECs from C57BL/6 mice (3–4 mice/prep) and bEnd5 cells (between passages 25–30) were pre-treated in serum-free condition for 16 h with recombinant hAng-2 (500 ng/ml) followed by AKB-9785 treatment (bEND5: 1, 10 or 50 µM; MBMECs: 50 µM) for 10 min. Data were analyzed by two-tailed unpaired *t* test for statistical significance.

### qPCR analysis

The SYBR^®^ Green system (Thermo Scientific) was used for qPCR analysis. The reaction of qPCR was performed in ABgene PCR plates (Thermo Scientific), sealed with a Clear Seal Diamond Heat Sealing Film (Thermo Scientific) and run in a CFX96™ Real-Time System (Bio-Rad) as described previously [15]. Using the reference gene, the relative expression of cDNA was calculated with CFX manager 3.1 software (Bio-Rad). The statistical analysis was performed by two-tailed unpaired *t* test. The primers used are listed in Table 3.

**Table 3:** Primers used for q-RT-PCR analysis

Primers	Sequence 5′–3′ sense	Sequence 5′–3′ antisense
hAng-2	agaggctgcaagtgctggaga	Ttgctccgctgtttggttca
Tie2	cctgggcctgtgagacgaat	Ccaggcactttgatgttctgct
CD31	attcctcaggctcgggtcttc	Ccgccttctgtcacctccttt
Mfsd2a	gggacggaaagttcacacggc	Agtcacgctcactctgctccg
Meca32	gactacgcgacgtgagatgga	aggatgatagcggcgatgaag

### Electron microscopy

CD1 mice (*n* = 3 for each genotype) were anesthetized and transcardially perfused with PBS for 1 min as described earlier followed by 4 min with 4 % PFA/2 % glutaraldehyde in PBS. The isolated brains were post-fixed with the same fixative ON at 4 °C. The tissue (cut into small pieces) was additionally post-fixed with 1 % OsO4 for 2.5 h at RT and stained with 2 % uranyl acetate solution at 4 °C ON prior to dehydration in graded acetone and embedded in Durcupan. The polymerization was performed at 60 °C for 72 h.

For the quantification of vesicles, 20 cortical vessels of comparable size were analyzed for each animal (*n* = 3 per genotype). For the presence of gaps within EC junctions, and for pericyte degeneration and pericyte coverage, 100 cortical vessels were analyzed from each brain (*n* = 5 for each genotype). Data were analyzed by one-sample *t* test as for gaps in EC junctions and Student’s unpaired *t* test for the rest.

To stain for the glycocalyx, mice (*n* = 3 each genotype) were anesthetized as described above and perfused with 2 % glutaraldehyde, 2 % saccharose, 0.1 M sodium cacodylate buffer and 2 % lanthanum nitrate (pH 7.4). Brains were isolated and fixed for 2 h in the perfusion solution and subjected to 2 % H_2_O_2_, 2 % saccharose, 0.1 M sodium cacodylate buffer and 2 % lanthanum nitrate for 12 h at 4 °C followed by rinsing with 0.03 M NaOH and 2 % saccharose. The contrast was increased by using a 2 % OsO_4_ and 2 % lanthanum nitrate followed by dehydration in graded ethanol and propylene oxide and embedding in araldite. The polymerization was performed at 60 °C for 12 h. For standard processing, ultrathin sections were contrast enhanced with uranyl-acetate and lead citrate. The analysis of glycocalyx was performed without any contrast enhancement to rule out an unspecific precipitation of the staining solutions. Sections were analyzed using Tecnai Spirit BioTWIN FEI electron microscope at 120 kV. Images were taken with an Eagle 4K CCD bottom-mount camera.

### HRP detection by electron microscopy

HRP tracer (type II, Sigma) was injected via tail vein into WT or Ang-2 GOF (10 mg/mouse) and allowed to circulate for 30 min before killing the mice. The brains were dissected and transferred into 5 % glutaraldehyde/4 % PFA/PB for 60 min at RT. After post-fixation in 4 % PFA/PB at 4 °C for 5 h, the brains were washed in PB at 4 °C ON. Serial coronal sections (50 µm) were cut on a Vibratome (Leica, VT1000S) and stained with 5 mg DAB/10 ml 0.05 M Tris–HCl, pH 7.6, containing 0.01 % H_2_O_2_ for 45 min at RT (DAB (3,3′-diaminobenzidine tetrahydrochloride, Sigma). Small regions of cortex were cut out and processed for electron microscopy as described previously. Shortly, sections were washed twice with PBS for 30 min. The tissue was then post-fixed with 1 % OsO_4_/1.5 % K_3_Fe(CN)_6_ for 2 h at RT, washed with dH_2_O followed by dehydration in graded acetone solutions and embedded in Durcupan. The polymerization was performed at 60 °C for 48 h.

Mice without HRP injection that were processed in the same way served as controls. For quantitation, vesicles and junctions of 30 vessels per brain were analyzed for the presence of HRP (*n* = 3 for each genotype). Data were analyzed by one-sample *t* test for HRP+ junctions and Student’s *t* test for the amount of HRP+ vesicles normalized to the total number of vesicles.

### Flow cytometry

Normal brains of adult Ang-2 GOF mice and wild-type littermates were dissected and immediately transferred in ice-cold HBSS. Briefly, following gentle mincing, the tissue was incubated in an HBSS solution containing Collagenase P (0.2 mg/ml), Dispase II (0.8 mg/ml), DNase I (0.01 mg/ml), Collagenase A (0.3 mg/ml) for 60 min at 37 °C under gentle rocking as previously described [52]. Following myelin removal, the cells were preincubated with rat anti-mouse FcγIII/II receptor (CD16/CD32) blocking antibodies (≤1 μg/million cells/100 μl; BD) for 5 min at 4 °C, and stained with the fluorochrome-conjugated antibodies (0.25–1 µg): CD45 PercP Cy5.5 (clone 30-F11, BD Biosciences), Gr-1 APC-Cy7 (clone RB6-8C5, BD Biosciences), CD11b APC (clone M1/70, BD), F4/80 FITC (clone BM8, eBioscence). Following two washing steps, DAPI (4′,6-diamidino-2-phenylindole; Invitrogen) was added for live gating and cells were acquired on a FACSCanto™ II flow cytometer (BD) using Diva Software (BD) and further analyzed using FlowJo analytical software (FlowJo Version 10.0.8, LLC). Background fluorescence levels were determined by Fluorescence Minus One (FMO).

## Results

**Fig. 1:**
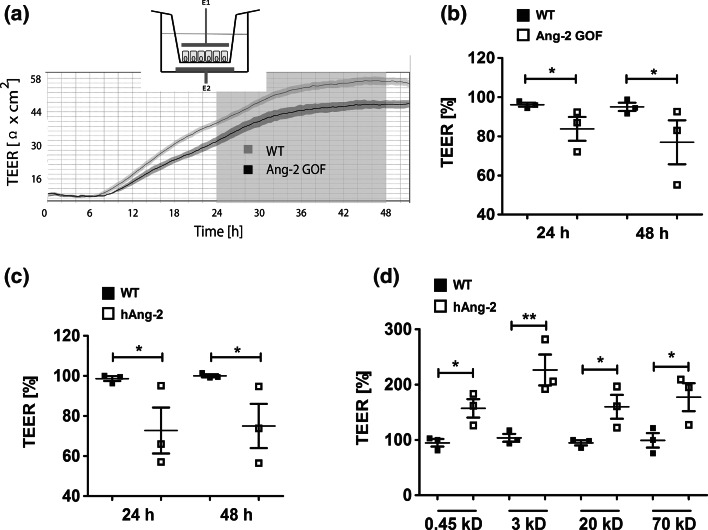
Ang-2 increases brain endothelial permeability in vitro. **a** MBMECs from WT and Ang-2 GOF brains were seeded on transwell inserts and transferred to a cellZscope^®^ system to obtain continuous TEER values. The *inset* shows a transwell insert along with top and bottom electrodes typical of a cellZscope^®^ device. The graph shows a representative experiment that indicates reduced TEER values in Ang-2 GOF MBMECs compared to WT that is sustained up to 48 h post-seeding when the monolayers reach confluency. **b** GOF MBMECs showed lower TEER than control cells at 24 and 48 h. **c** WT-MBMECs treated with hAng-2 resulted in reduced TEER. **d** hAng-2 treatment resulted in increased tracer flux across MBMECs (**b–d**, *n* = 3)

**Fig. 2:**
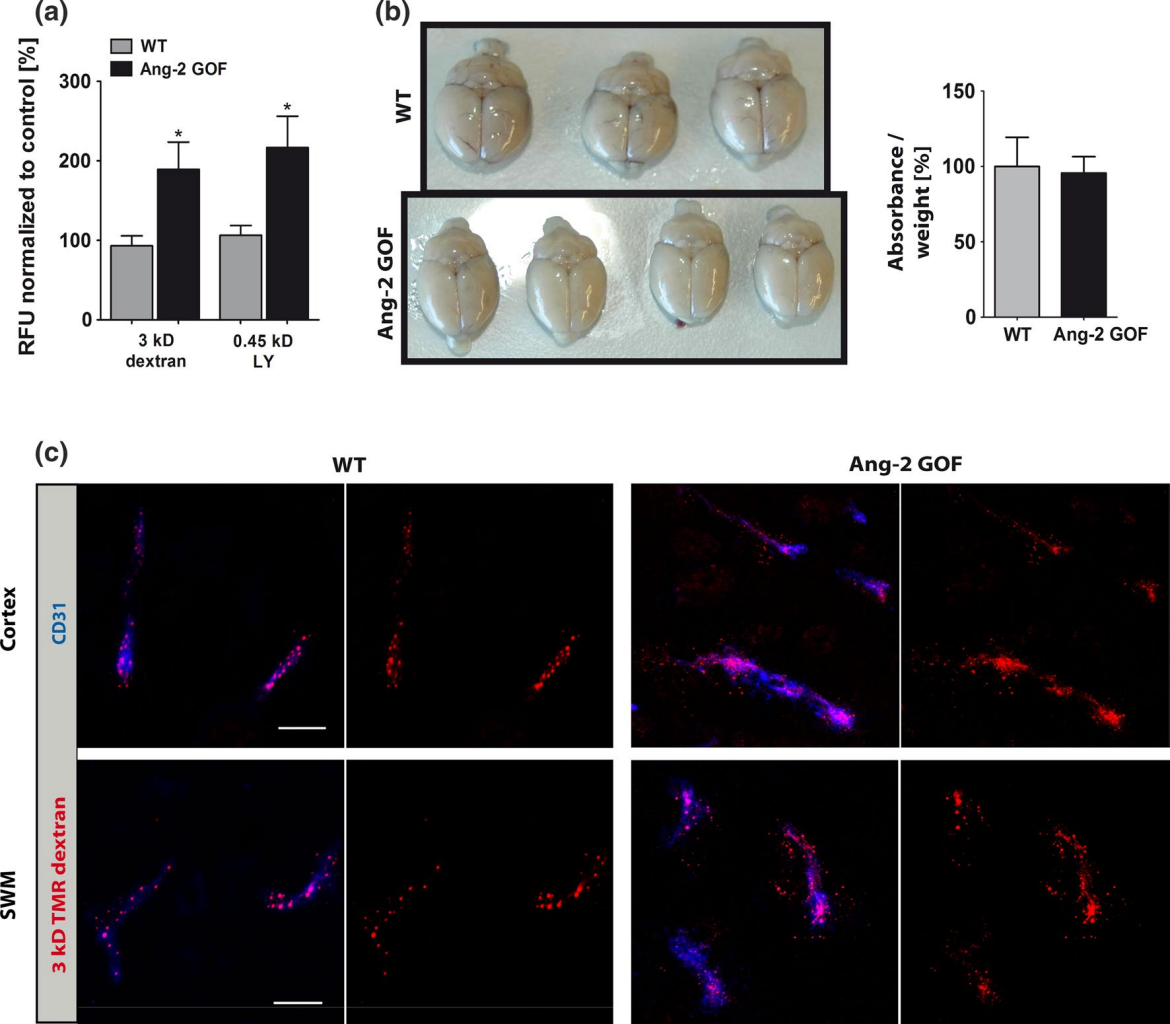
Permeability analysis of Ang-2 GOF mice in vivo. **a** Permeability to LY and Texas Red-3 kD/TMR-dextran was higher in GOF brains (LY: *n* = 7, TXR: WT *n* = 12; GOF *n* = 16). **b** Evans blue dye permeability was not altered (*n* = 6). **c** Increased permeability of 3 kD TMR-dextran in Ang-2 GOF mice in cortex and subcortical white matter (SWM) (*n* = 3, *scale bars* 10 µm)

The effect of Ang-2 on BBB permeability was first assessed by transendothelial electrical resistance (TEER) and capacitance (Ccl) measurements on the primary mouse brain endothelial cells (MBMECs) (Fig. 1a). The TEER of MBMECs derived from Ang-2 GOF mice was lower compared to WT (Fig. 1b), and Ccl was higher (Suppl. Fig. 1a) indicating a barrier-opening effect of Ang-2. Similar results were obtained when WT-MBMECs were treated with recombinant Ang-2 (hAng-2, Fig. 1c). The permeability to fluorescent dextrans (3, 20, and 70 kD) and Lucifer Yellow (LY 0.45 kD) [15] was also increased in TEER measurement by hAng-2 (Fig. 1d), suggesting a direct effect of Ang-2 on brain EC permeability. In vivo, the permeability to LY and 3 kD dextrans (Texas Red and TMR) was significantly increased in Ang-2 GOF (Fig. 2a, c), with no difference for Evans blue dye (~70 kD) potentially owing to a tight barrier in vivo (Fig. 2b). The 0.45 kD LY and 3 kD dextran tracers were circulated for 4 min due to their rapid clearance from blood vessels at longer time points (Suppl. Fig. 1b). Kidneys served as a control for tracer permeability that was not altered between GOF and WT (Suppl. Fig. 1c). These results therefore demonstrate Ang-2-mediated brain endothelial permeability for low molecular weight solutes in vivo.

**Fig. 3:**
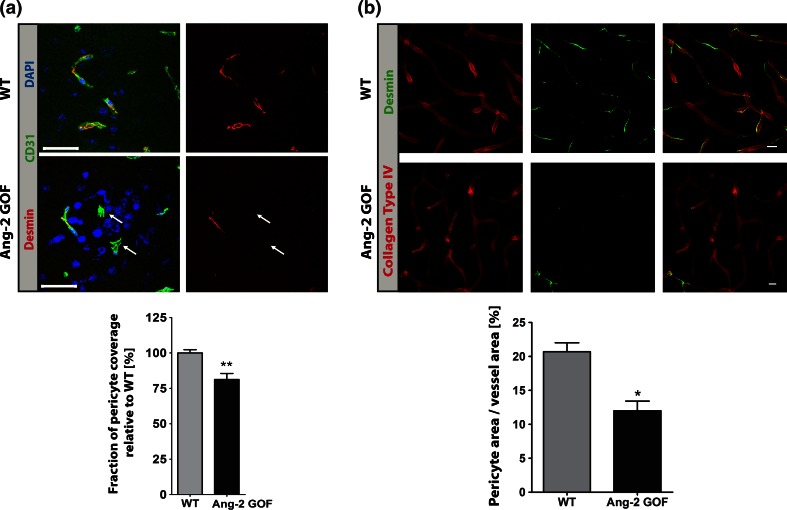
Immunohistochemistry analysis of pericytes in Ang-2 GOF mice. **a** Pericytes (desmin+) were decreased in GOF mice utilizing 10 µm cryosections for analysis (*n* = 8, *scale bars* 25 µm). **b** 50 µm vibratome sections of Ang-2 GOF and WT mice revealed decrease in pericyte area/vessel area [%] (*n* = 3, *scale bars* 10 µm) indicating decreased pericyte coverage of the vessels in GOF mice

**Fig. 4:**
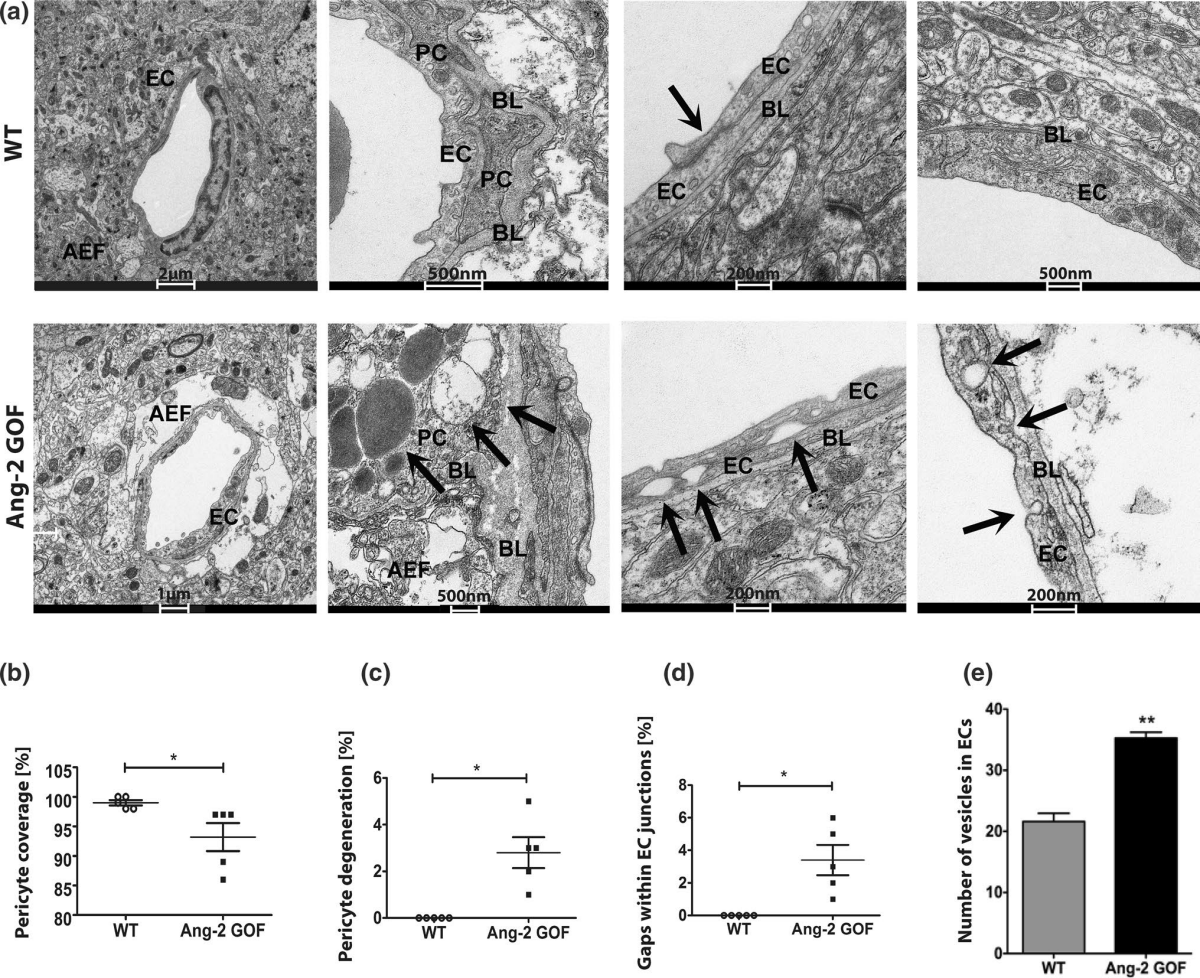
Ultrastructural analysis of Ang-2 GOF mice. **a** EM revealed swollen astrocytic endfeet, degenerating pericytes and more vesicles and gaps (*arrows*) between ECs in GOF. *AEF* astrocytic endfeet, *BL* basal lamina, *PC* pericyte (*scale bar* sizes are indicated in the images). **b** The pericyte coverage analyzed from EM images is decreased in Ang-2 GOF mice (100 vessels were analyzed; *n* = 5). **c** Increased pericyte degeneration was observed in Ang-2 GOF mice (100 vessels analyzed; *n* = 5). **d** The gaps within EC junction increased in Ang-2 GOF mice (100 vessels analyzed; *n* = 5). **e** The number of cortical vesicles of ECs in Ang-2 GOF mice increased (20 vessels of similar size 4–5 µm lumen per mouse were analyzed; *n* = 3)

**Fig. 5:**
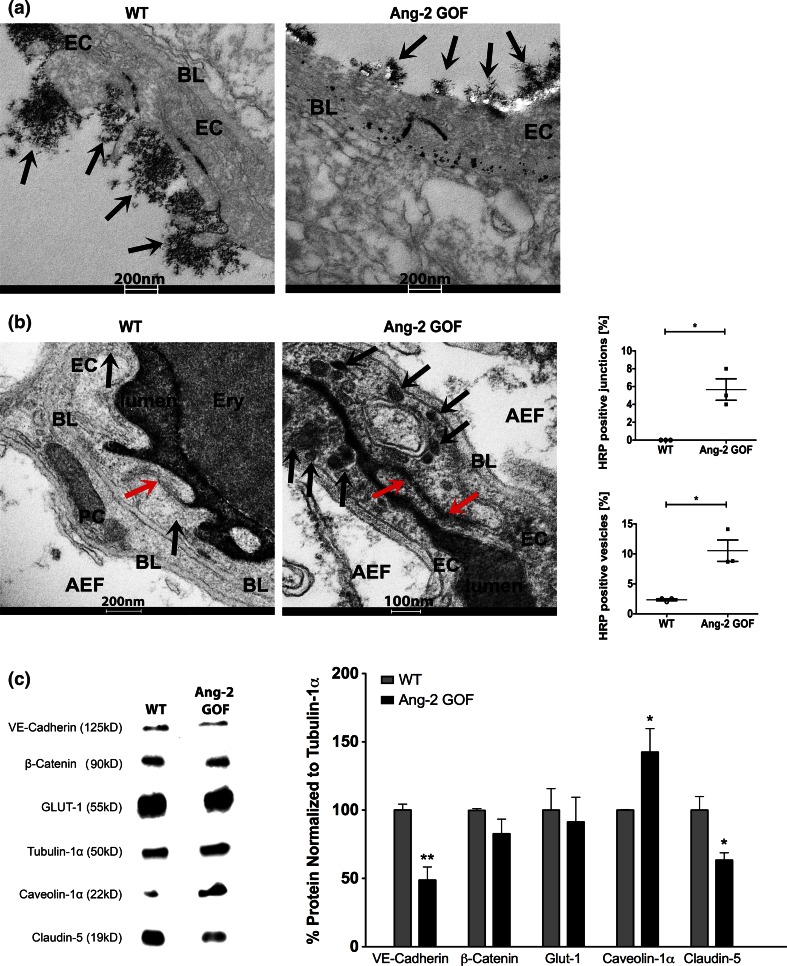
Detection of the glycocalyx and plasma tracers by EM analysis: **a** lanthanum nitrate was used to detect the glycocalyx (*black arrows*) in mouse brain vessels. Glycocalyx was decreased/disrupted from ~300 to ~100 nm in GOF mice. **b** HRP was intravenously injected into WT and Ang-2 GOF mice and circulated for 30 min. Representative images of Ang-2 GOF mice revealed HRP-vesicles (*black arrows*) with affected endothelial junctions (*red arrows*). Quantitative analysis indicated more HRP-positive vesicles in Ang-2 GOF mice (20 vessels analyzed; *n* = 3; *scale bar* sizes are indicated in the images). **c** MBMV Western blots showed decreased VE-cadherin and claudin-5 in GOF, whereas caveolin-1 was upregulated (*n* = 3)

We next elaborated the molecular components of Ang-2-mediated permeability by immunohistochemistry (IHC), electron microscopy (EM) and Western blots. Quantitative RT-PCR analysis confirmed an overexpression of hAng-2 in the isolated brain microvessels of Ang-2 GOF mice, whereas Tie2 expression levels were not altered (Suppl. Fig. 1d). Furthermore, we did not observe an increase in Ang-1 levels (Suppl. Fig. 1e, f) in these mice, suggesting an absence of its compensatory effects. IHC from cryosections (10 µm) and from vibratome sections (50 µm) revealed a decrease in pericytes in GOF (Fig. 3a, b; Suppl. video) as previously observed in the periphery [53]. In Fig. 3a, we set the WT pericyte coverage to 100 %; however, the absolute pericyte coverage of the vessels is only about 20–25 % as obtained from vibratome data analysis which is reduced to approximately 12–15 % in the GOF mice. Also by EM analysis, we observed decreased pericyte coverage with increased numbers of degenerating pericytes in Ang-2 GOF mice (Fig. 4a–c; Suppl. Table 1). EM analysis also showed increased caveolae-like vesicles (Fig. 4a, e) that are partly responsible for peripheral endothelial permeability. The analysis also revealed decreased complexity of the inter-endothelial junctions in GOF, with frequent gaps (Fig. 4a, d; Suppl. Fig. 2a; Suppl. Table 2), indicating increased paracellular permeability. Furthermore, the glycocalyx thickness was considerably disrupted/decreased (~300–~100 nm, Fig. 5a; Suppl. Fig. 2b). This finding implies increased permeability induced by Ang-2 and is in line with a previous report suggesting that in contrast to Ang-2, Ang-1 increases the glycocalyx depth and reduces vascular permeability [31, 49]. To further elaborate the permeability in GOF mice, we analyzed the permeability of circulating HRP tracer by EM. We observed a greater number of HRP-positive vesicles in Ang-2 GOF mice compared to WT mice (Fig. 5b; Suppl. Table 3). These data indicate that Ang-2 could lead to transcellular vesicular permeability and are in line with previous work showing increased caveolae-mediated transport upon pericyte loss and subsequent permeability in genetic mouse models [4]. Western blots of isolated mouse brain microvessels (MBMV) revealed a downregulation of VE-cadherin and claudin-5 in GOF animals, whereas caveolin-1, crucial for caveolae formation was upregulated (Fig. 5c). These data support the EM data that indicated increased numbers of vesicles and gaps in junctions (Figs. 4, 5b; Suppl. Fig. 2a). Glut-1 and β-catenin were however unaltered (Fig. 5c). However, mRNA levels of MECA 32, a plasmalemma vesicle-associated protein (PLVAP), and Mfsd2a that is associated with increased vesicular formation upon pericyte degeneration, were not altered (Suppl. Fig. 4a). Further, flow cytometry analyses revealed an increased number of myeloid cells in brains derived from Ang-2 GOF mice compared to WT mice (Suppl. Fig. 3a), a finding that is consistent with previous observations [52]. Together, these data indicate an effect of Ang-2 on both paracellular and transcellular permeability.

Astrocytes play an essential role in BBB permeability [2]. Aquaporin-4 IHC did not show any change in the astrocytic endfeet coverage of GOF brain vessels (Suppl. Fig. 3b). However, EM analysis revealed astrocytic endfeet swelling (Fig. 4a) and occasional pericyte degeneration (Fig. 4a, c; Suppl. Table 1), also indicative of neurovascular uncoupling. These results together suggest that Ang-2 increases BBB permeability by modulating both paracellular junctions and transcellular vesicular transport systems.

**Fig. 6:**
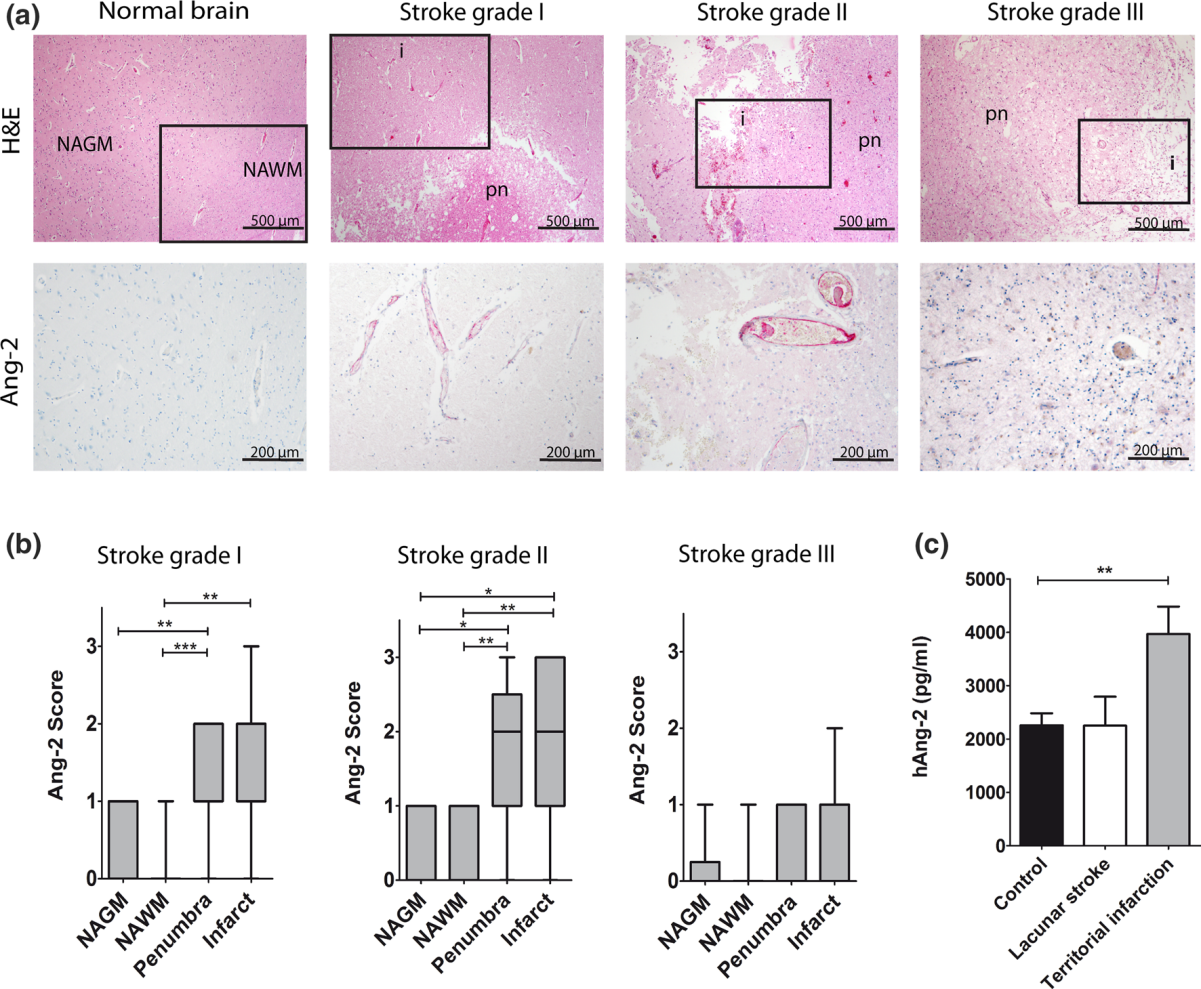
Ang-2 expression analysis in normal brain and human stroke samples (grade I–III). **a**, **b** H&E/IHC staining of human stroke samples showed higher Ang-2 expression in the stroke area (*n* = 13). *NAWM* normal appearing white matter, *NAGM* normal appearing gray matter, *i* infarct, *pn* penumbra. **c** Serum ELISA from stroke (*n* = 4-lacunar, *n* = 15-territorial) and healthy subjects (*n* = 16) showed highest Ang-2 levels in territorial strokes

**Fig. 7:**
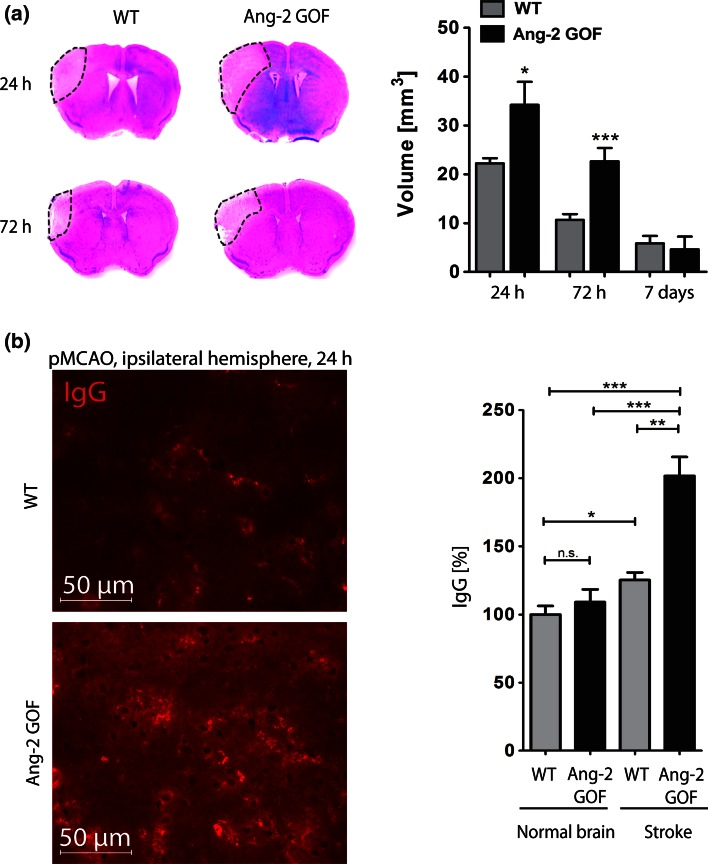
Analysis of stroke severity in Ang-2 GOF mice. **a** pMCAO infarcts were larger in GOF (24 h, WT, GOF *n* = 4; 72 h, WT *n* = 10, GOF *n* = 7; 7 days, WT, GOF *n* = 5). **b** IgG permeability in the infarct area was higher in GOF (*n* = 6) compared to WT (*n* = 4)

The above-mentioned findings prompted us to investigate the potential role of Ang-2 in human cerebrovascular disease. Expression levels of Ang-2 were assessed by IHC in human brain autopsy specimens and by ELISA in stroke patients compared to healthy volunteers. While Ang-2 expression was almost absent in normal brain tissue, the levels were higher in stroke grades I and II that are characterized by BBB leakage (Fig. 6a, b) and returned to low expression levels in grade III lesions (Fig. 6a, b). Serum analysis from stroke patients indicated a significantly higher level of hAng-2 in patients with territorial stroke compared to healthy humans (Fig. 6c). We subsequently employed a mouse permanent middle cerebral artery occlusion (pMCAO) model, where significantly larger infarcts were detected in Ang-2 GOF mice after 24 and 72 h compared to WT mice (Fig. 7a), whereas at 7 days post-stroke differences in infarct size between WT and GOF resolved. Additionally, the IgG permeability in the stroke area was increased in GOF mice compared to WT 24 h after occlusion (Fig. 7b) corroborating the permeability effects of Ang-2 in a stroke model.

**Fig. 8:**
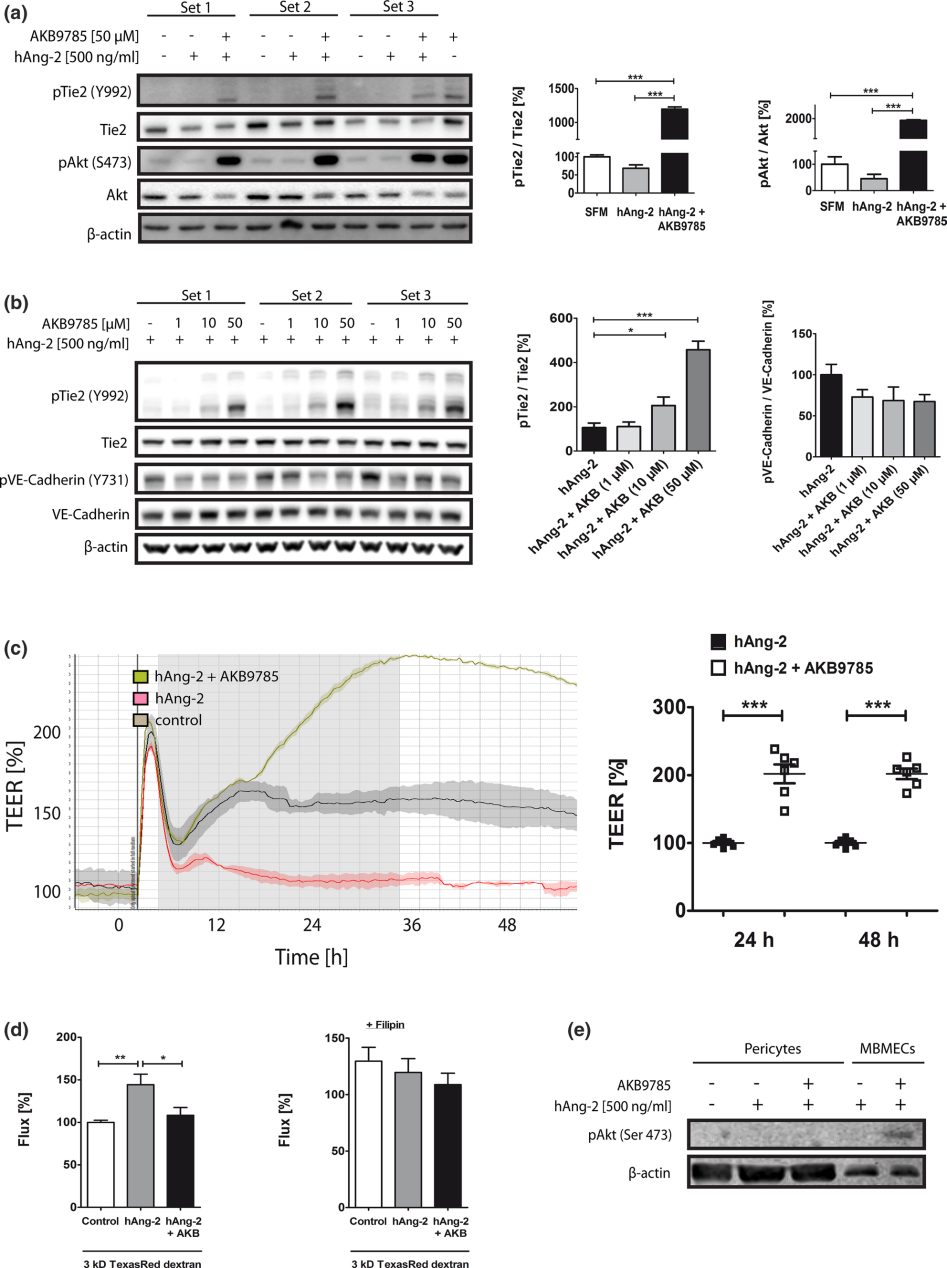
Effects of AKB-9785 treatment in vitro. **a** MBMECs pre-treated with hAng-2 increased Tie2/Akt activation upon AKB-9785 treatment (*n* = 3). **b** bEnd5 Western blots revealed Tie2/Akt activation, but a decrease (non-significant) in pVE-cadherin by AKB-9785 (*n* = 3). **c** MBMECs pre-treated with recombinant hAng-2 [500 ng/ml] followed by AKB-9785 [1 µM] treatment revealed increased TEER values. **d** The in vitro permeability tracer experiment with a 3 kD Texas Red-dextran showed a decrease in permeability with AKB-9785 + hAng-2 compared to hAng-2 treatment alone. This difference was not detected in the presence of filipin. **e** The phosphorylation of Akt in Western Blot analysis could only be identified in MBMECs, but not pericytes (*n* = 3)

**Fig. 9:**
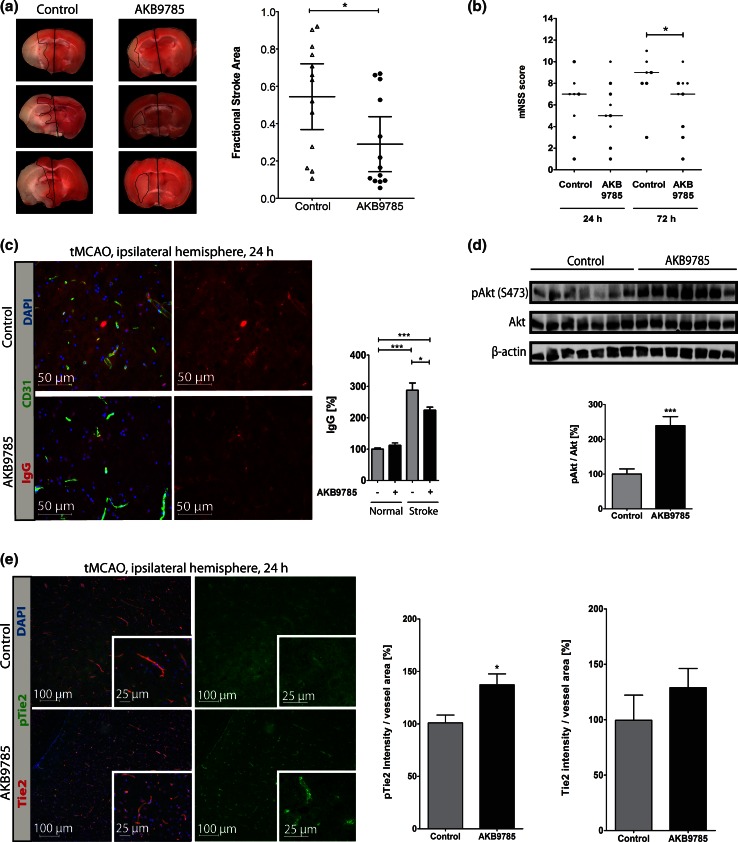
Therapeutic targeting of Tie2 signaling using AKB-9785 in WT mice subjected to tMCAO. **a** TTC staining after tMCAO (3/group) showed significantly smaller infarcts 24 h post-stroke upon AKB-9785 treatment (*n* = 13). **b** An mNSS behavioral analysis of control and mice treated with AKB-9785 (30 mg/kg) showed a neurological improvement in treated mice 72 h after stroke incidence (24 h: control *n* = 8, AKB-9785 *n* = 9; 72 h: control *n* = 7, AKB-9785 *n* = 9). The neurological deficit scoring is described in “Materials and methods” and is composed of testing flexion, gait, coordination and sensory functions. The total scoring included all four behavioral tests (2-tailed unpaired non-parametric Mann–Whitney test). **c** IgG staining showed significantly lower permeability in AKB-9785 treated mice (*n* = 6). **d** Western blots of stroke hemisphere showed increased Akt activation in AKB-9785-treated mice (*n* = 7). **e** Tie2/pTie2 staining of the stroke hemisphere revealed significantly higher pTie2, but not total Tie2 levels in AKB-9785-treated animals (*n* = 5)

We had previously observed increased Ang-2 levels in WT rodents subjected to MCAO [7] and thus sought to investigate the rescue of stroke size/permeability in WT mice by interference with Ang-2/Tie2 signaling. To this end, we employed the more clinically relevant transient MCAO (tMCAO) model in WT mice and aimed a rescue with AKB-9785 (gift from Aerpio Therapeutics), a drug targeting Tie2 signaling by blocking the activity of the vascular endothelial protein tyrosine phosphatase (VE-PTP) that is associated with Tie2 and VE-cadherin [54]. In vitro, MBMECs/bEnd5 pre-treated with hAng-2 followed by AKB-9785 treatment showed an increase in pTie2 and pAkt, but not in pVE-cadherin (Fig. 8a, b), associated with decreased junctional permeability as shown by increased TEER of MBMECs after 24 and 48 h (Fig. 8c) as well as decreased capacitance (Ccl) values (Suppl. Fig. 4b). The permeability effect of hAng-2 decreased in combination with AKB-9785 (Fig. 8c, d). Upon filipin treatment, a compound blocking the formation of caveolae, no differences in 3 kD dextran tracer between control, hAng-2 and hAng-2 plus AKB-9785 could be detected anymore (Fig. 8d). These data support our hypothesis that hAng-2 causes permeability partly by transcellular vesicular permeability and that AKB-9785 is able to prevent this effect. In the 67 kD BSA-Alexa 647 tracer, this effect was abolished, suggesting that Ang-2 induced BBB permeability primarily facilitates transition of smaller molecules (Suppl. Fig. 4c). The effect of AKB-9785 is endothelial specific, as phosphorylation of Akt could neither be detected in pericytes (Fig. 8e) nor in astrocytes (Suppl. Fig. 4d). WT tMCAO mice treated with AKB-9785 displayed reduced infarcts (Fig. 9a) and permeability to IgG (Fig. 9c) with a post-stroke neurological improvement 72 h after occlusion (Fig. 9b). The AKB-9785-treated hemispheres from mice subjected to tMCAO showed increased pTie2/pAkt activity (Fig. 9d, e). This finding is indicative of a restoration of the Tie2 signaling as pTie2 staining in AKB-9785-treated stroke brains appeared almost similar to pTie2 staining in normal brain vessels (unaffected hemispheres; Suppl. Fig. 5, compare to Fig. 9e). We next ascertained whether the increased permeability upon stroke in Ang-2 GOF mice was a direct effect of Ang-2 or secondary to stroke that is associated with impaired BBB. To this end, we performed permeability analyses at 3 h post-stroke, a time point associated with minimal infarct sizes. The results revealed already increased extravasation of 3 kD TMR-dextran in Ang-2 GOF mice 3 h post-stroke suggesting that Ang-2-mediated permeability could contribute to edema formation and thus facilitate larger infarct size at later time points (Suppl. Fig. 4e). These results are further supported by the therapeutic rescue achieved by administration of AKB-9785, potentially reducing BBB leakage by VE-PTP-dependent activation of Tie2 signaling.

## Discussion

Brain endothelium is the principal site of BBB regulation, which largely restricts the access of plasma components to the brain parenchyma. The BBB is disturbed in many neurological disorders resulting in increased cerebrovascular leakage and brain edema [16, 56]. It is well established that Ang-2 influences permeability in peripheral vascular beds [11, 64]; however, its role in regulating the permeability of specialized brain endothelium is still unclear and was thus the focus of the present study. Our results demonstrate that endothelial cell-derived Ang-2 is able to increase brain endothelial permeability in vitro and in vivo and is associated with increased stroke size. Activating Tie2 signaling by an inhibitor of VE-PTP partly reversed cerebrovascular permeability and increased neurological function in a mouse stroke model.

In our previous work, we showed that Ang-2 GOF in ECs leads to a loss of pericytes in the peripheral vessels [53], an effect which was likewise observed in other studies and associated with an increase in endothelial permeability [64]. In conjunction with the established role of pericytes in regulating the permeability of BBB [4, 10, 18], we thus aimed to investigate whether Ang-2 also affects the permeability of the BBB. To this end, we utilized electrical impedance measurements to obtain continuous barrier tightness indices of the primary cultured mouse brain endothelial cells (MBMECs). Our results established that TEER of MBMECs was decreased upon recombinant Ang-2 treatment or upon Ang-2 GOF, indicative of a barrier-opening effect of Ang-2. We also observed increased capacitance (Ccl) values in Ang-2 GOF MBMECs, reflective of an increase in transcellular permeability as capacitance is a property of the cellular membranes in storing charges (discussed in detail in [15]). However, as TEER and Ccl are indicative of permeability to ions, we extended the Ang-2-mediated permeability of MBMECs to other solutes [15] and observed that Ang-2 increased permeability to tracers of different sizes.

To confirm the observed permeability effects of Ang-2 on brain endothelium in transgenic mice in vivo, we performed fluorescent tracer studies. For these experiments, we specifically chose to employ endothelial-specific Ang-2 GOF mice as compared to recombinant Ang-2 treatment, as the former is specific to the effects arising from ECs, the principal site of Ang-2 expression. We initially tested the leakage of large dyes such as Evans blue (~70 kD) in mice that express transgenic Ang-2 in EC. There was no difference in Evans blue dye leakage between WT and GOF mice indicating that the barrier-opening effects of Ang-2 in vivo to bigger tracers was lower than that in vitro where 70 kD FITC-dextran permeability was increased twofold. However, the permeability to LY and 3 kD TXR/TMR-dextran was increased in Ang-2 GOF mice in vivo. Thus, our results clearly demonstrate that endothelial-derived Ang-2 regulates the size-based permeability at the healthy BBB. Our observations extend previous findings on permeability-modulating functions of Ang-2 in peripheral vascular beds [11, 64] to the highly specialized endothelium at the BBB. As Ang-1 and Ang-2 have an established role as antagonizing molecules, our findings are additionally in line with former reports on barrier-tightening effects of Ang-1 in the skin of genetically engineered mice [59, 60]. The size-based permeability in vivo most likely reflects an extremely tight barrier that, however, is compromised in brain EC monolayer cultures in vitro [20]. We hypothesize that the differences in Evans blue dye versus smaller sized tracers may be due to the size-based permeability arising from gaps in EC junctions. It could also arise due to differences in diffusion time that is a function of the tracer size and route—paracellular versus transcellular, which is mediated by vesicles and potentially receptor-based transport. Moreover, the size-restricted permeability could also be a result of greater clearance of the Evans blue dye from circulation at later time points than observed for smaller tracers. To address this issue in more detail, we assessed regional differences in permeability by immunofluorescence of injected tracers using anti-dye antibodies to enhance the signal. In two regions investigated, namely cortex and subcortical white matter (SWM), we observed an increase in 3 kD dextran extravasation in Ang-2 GOF mice, as indicated by extravascular dye staining and a loss of vascular staining. In contrast, the dye remained restricted to the vessels in the WT mice.

The molecular components of the Ang-2-mediated increase in endothelial permeability was elaborated by immunohistochemistry (IHC) and ultrastructural analysis (electron microscopy) of the neurovascular unit in Ang-2 GOF mice and by biochemical analyses of isolated microvessels. It has previously been reported that astrocytic endfeet coverage has an essential role in barrier permeability [30, 35], and more recently it was shown that pericytes are essential for maintaining low BBB permeability by preventing caveolae-mediated transcytosis [4]. Our results do not directly parallel the former findings, because we did not observe changes in astrocytic endfeet coverage in Ang-2 GOF as demonstrated by IHC with the astrocyte endfeet marker aquaporin-4. However, our EM analysis indicated neurovascular uncoupling as inferred from astrocytic endfeet swelling as well as pericyte degeneration. Apart from this, an Ang-2-mediated effect on peri-vascular cells in brains of Ang-2 GOF mice is indicated by decreased pericyte coverage, which is in line with previous finding of pericyte loss in peripheral vessels of the GOF mice [53]. Our EM analysis further indicated increased flask-shaped plasmalemmal vesicles in Ang-2 transgenic mice reminiscent of caveolae, a subset of the lipid rafts that are responsible for higher endothelial permeability of peripheral vessels [13]. These results are supported by findings using HRP, a plasma tracer that was injected intravenously followed by EM. Our results clearly evidenced higher numbers of HRP-positive vesicles in Ang-2 GOF when normalized to the total number of vesicles, indicative for transcytosis of blood-borne tracers via vesicles in Ang-2 GOF mice. These data are in line with previous work showing an increase in caveolae-mediated transport upon pericyte loss and subsequently enhanced permeability [4]. The EM analysis also revealed the decreased length of the inter-endothelial junctions with frequent gaps indicative of increase in paracellular permeability mediated by Ang-2. To gain further insight on brain vessel morphology at the BBB, we investigated the luminal glycocalyx size in Ang-2 GOF and WT mice, as it had previously been established that the glycocalyx influences vascular permeability via its binding to plasma proteins [55]. By means of lanthanum nitrate staining, we demonstrated that the glycocalyx in GOF mice was considerably decreased and disrupted in all vessels. Furthermore, we observed particles of lanthanum nitrate on the abluminal side deposited in the basement membrane, which were occasionally found in intracellular vesicles suggesting transcytosis of glycocalyx fragments. Although it is currently unclear how Ang-2 mediates the shedding of glycocalyx, our ultrastructural analyses clearly substantiated the disrupted barrier functions in Ang-2 GOF mice that ultimately led to increased permeability.

We performed further biochemical analyses on mouse brain microvessels to assess the involvement of permeability-related molecules, both junctional and those involved in transcellular transport. The analyses revealed the downregulation of VE-cadherin, a crucial adherens junction molecule, as well as claudin-5, a tight junction molecule. The obtained results fully support our observations at the ultrastructural level that revealed gaps in endothelial junctions. Furthermore, caveolin-1, a scaffolding protein crucial for caveolae formation, was upregulated, once again reassuring EM data that showed increased caveolae-like vesicles in the Ang-2 GOF mice. We however did not observe any change in Glut-1, a transendothelial facilitative glucose transporter, suggesting that specific modulations of junctional and vesicular proteins caused by Ang-2 result in the permeability increase of the brain microvasculature. Our study consequently demonstrates that Ang-2 increases both the paracellular and the transcellular permeability at the BBB.

These findings may have clinically relevant implications for neurological disorders that are associated with BBB disturbance and cerebral edema, including hemorrhagic and ischemic stroke, traumatic brain injury, epilepsy, neurodegenerative disease, high-altitude disease as well as primary and metastatic brain neoplasia [16, 56]. Of note, Ang-2 has been shown to be upregulated in the activated endothelium in stroke and glioblastoma models in rodents as well as in glioblastoma patients [7, 32, 52, 57], suggesting that therapeutic interference with Ang-2/Tie2 signaling may be particularly useful to prevent BBB disruption in those disorders. The successful targeting of Ang-2-mediated BBB breakdown has previously been emphasized in preclinical models of orthotopic glioma and breast cancer metastasis to the brain [6, 52]. In our study, we specifically aimed to interfere with the BBB breakdown in mouse models of cerebral ischemia. Stroke is a leading cause of death and the most common cerebrovascular disorder, characterized by an initial breakdown of the BBB with increased edema into the surrounding brain parenchyma [8, 50]. We have previously demonstrated increased endothelial-derived Ang-2 levels in a rodent experimental ischemic stroke model [7] and in the present study therefore investigated the potential role of Ang-2 in stroke patients. Indeed, similar to our findings in the rodent stroke models, autopsy cases revealed increased Ang-2 expression levels in stroke grade I and II samples. As expected, in stroke grade III that is characterized by cysts, glial scar tissue and a largely recovered BBB function, Ang-2 went back to normal levels. Interestingly, Ang-2 serum levels were significantly higher in patients with large, territorial infarcts compared to serum levels in healthy volunteers. However, patients with smaller, lacunar infarcts had levels more similar to healthy volunteers. These findings are in line with previous reports identifying Ang-2 as a crucial risk factor in a variety of cardiovascular diseases, including myocardial infarction, and thus suggest that Ang-2 serum levels might in addition serve as useful biomarkers to assess endothelial dysfunction in stroke patients [33, 38].

To more directly assess the impact of Ang-2 in stroke at the molecular level, a permanent MCAO stroke model was employed in Ang-2 GOF mice. Our data further support the involvement of Ang-2 in stroke pathophysiology, since we observed larger infarcts and increased permeability in GOF mice at 24 h that were also present at 72 h post-stroke albeit to a lower extent, potentially due to a greater BBB recovery at the later time point [48]. At 7 days post-stroke when BBB function is significantly recovered, no differences were observed in stroke size between the WT and GOF mice. To elaborate the permeability effects of Ang-2 in settings of cerebrovascular disease, we performed tracer experiments with a small molecular weight tracer (3 kD dextran) in the GOF mouse model. While dextran was mostly restricted to vessels in the WT mice 3 h post-stroke, extravascular dye was detected in the GOF mice. Our results strongly correlate with the clinical findings and implicate Ang-2 as an important mediator of permeability/BBB breakdown upon cerebral ischemia in mice.

Based on these findings, we explored the therapeutic rescue of experimental stroke in mice using an inhibitor of the vascular endothelial protein tyrosine phosphatase (VE-PTP), a pivotal negative regulator of Tie2 signaling [54]. Therapeutic targeting of VE-PTP in retinal/choroidal vascular diseases was emphasized by Shen et al. [54], as interference with VE-PTP signaling via AKB-9778 stabilized the ocular vasculature, even in the presence of high amounts of endogenous Ang-2. Pharmacological VE-PTP inhibition using the same inhibitor led to tumor vessel normalization through Tie2 activation and delayed tumor growth as well as metastatic progression [29]. VE-PTP also associates with VE-cadherin, whereby it supports the adhesive activity of VE-cadherin and the integrity of endothelial cell connections [12, 42]. Consistent with the role of VE-PTP as a negative regulator of Tie2 activation, our results clearly demonstrate that both pTie2 and pAkt were increased, whereas VE-cadherin was decreased upon AKB-9785 treatment [27]. Our findings thus imply that AKB-9785 activates Tie2 signaling at the BBB, independent of VE-cadherin phosphorylation. Importantly, this finding is in line with recent observations in a lung leukocyte transmigration model [27]. The authors of this study elegantly demonstrated that interference with VE-PTP stabilizes endothelial junctions in non-brain endothelial cells via Tie2 in the absence of VE-cadherin [27]. Our results demonstrate that AKB-9785 was able to rescue Ang-2-mediated permeability effects in vitro supporting its BBB tightening effects. Furthermore, Ang-2-mediated increase in permeability was not present after treatment with filipin, a compound that is known to disrupt caveolae, indicating that Ang-2 affects permeability by paracellular and vesicular pathways. To explore the potential therapeutic effects, AKB-9785 was evaluated in a transient MCAO model that is more reflective of human stroke pathophysiology than the previously employed permanent MCAO model. The effects of the inhibitor were assessed 24 h post-MCAO, a time point with pronounced BBB damage [48]. We observed a diminished stroke size compared to the control group with a concomitant neurological improvement after 72 h. This was accompanied by a decrease in permeability in the stroke area of treated animals. Activation of Tie2 signaling in vivo was supported by IHC showing the restoration of pTie2 staining in AKB-9785-treated MCAO brains. This finding is further supported by Western blot analysis showing Akt activation in AKB-9785-treated stroke samples, as well as primary brain EC. AKB-9778, a compound closely related to AKB-9785, was previously shown to have similar anti-permeability effects via Tie2 pathway and independent of the adhesive activity on VE-cadherin [27, 29, 54]. As such, the VE-PTP inhibitor AKB-9785 restores pTie2 to levels that ensure proper vascular function, consistent with the finding that pTie2 is constitutively expressed in the normal adult rodent brain [62]. Further, the current analysis indicates that the specific effects of the VE-PTP inhibitor AKB-9785 in improving stroke outcome are mediated solely by restoring Tie2 signaling and are independent of VE-cadherin phosphorylation.

The permeability of the BBB was also shown to be regulated by Wnt/β-catenin signaling effecting both the brain vascular development and maintenance of barrier properties [37]. Sonic hedgehog signaling has also been demonstrated to contribute to barrier maturation [3] and, more recently, the retinoic acid signaling has been shown to be a novel regulator of BBB function [41]. It is, however, not known if the above pathways are interacting in controlling BBB function. In this regard, it was recently demonstrated that shear stress increased Ang-2 expression via activation of the canonical Wnt pathway in aortic endothelial cells, an effect that was reversed with Wnt inhibition [36]. Similar findings were also observed in vascular repair in zebrafish. However, the authors did not assess the role of Ang-2 in inhibiting Ang-1-mediated Tie2 signaling which has an established role in maintaining vessel integrity [60]. Such a role for Ang-2 in vascular repair is supported by previous studies showing vascular regression in the absence of VEGF [32] and our work showing blood–brain barrier-stabilizing effects of canonical Wnt pathway [37]. The role of sonic hedgehog (shh) pathway in regulating angiopoietins has been reported in vitro in NIH 3T3 cells that do not express shh protein [34]. It was demonstrated using recombinant shh that resulted in the activation of target gene Gli1 and also led to the upregulation of Ang-1 mRNA and downregulation of Ang-2 mRNA. This effect was reversed in the loss of function experiments using cyclopamine, a specific inhibitor of the shh signaling pathway. The above role of shh in regulating Ang-1/Ang-2 is well supported by the established vessel maturation and barrier maintenance roles of the shh pathway [3]. Our finding that Ang-2 signaling plays a role in brain vascular permeability opens up the emerging question whether angiopoetin/Tie-2 signaling acts independently or in conjunction with other pathways.

In summary, we demonstrated that Ang-2 is a critical mediator of cerebrovascular permeability in vivo as evidenced in Ang-2 GOF mice and in models of experimental stroke. We further showed larger infarcts in Ang-2 GOF mice that corroborate data obtained in humans where the stroke grade and size correlated with Ang-2 expression levels. The Ang-2-mediated effect in stroke models was rescued by targeting Tie2/Akt signaling in a VE-PTP-dependent manner. We conclude that Ang-2 is a predominant regulator of BBB permeability and that targeting the angiopoietin/Tie2 signaling could lead to clinically relevant therapeutics for tightening the BBB in neurological disorders as shown here for ischemic stroke by employing a novel VE-PTP inhibitor. Vice versa, modulating Tie2 signaling could potentially be used for opening the BBB to facilitate drug delivery to the CNS.

## Electronic supplementary material

Below is the link to the electronic supplementary material.

Supplementary material 1 (TIFF 66519 kb)

Supplementary material 2 (TIFF 25692 kb)

Supplementary material 3 (TIFF 65399 kb)

Supplementary material 4 (TIFF 69086 kb)

Supplementary material 5 (TIFF 30487 kb)

Supplementary material 6 (DOCX 35 kb)

Supplementary material 7 (AVI 2883 kb)

Supplementary material 8 (AVI 2309 kb)
