# Supplementary figures and images for: Angiopoietin-2-induced blood–brain barrier compromise and increased stroke size are rescued by VE-PTP-dependent restoration of Tie2 signaling
Source: Acta Neuropathol. 2016 Mar 1;131:753–73. doi: 10.1007/s00401-016-1551-3 (PMC4835530; doi:10.1007/s00401-016-1551-3)

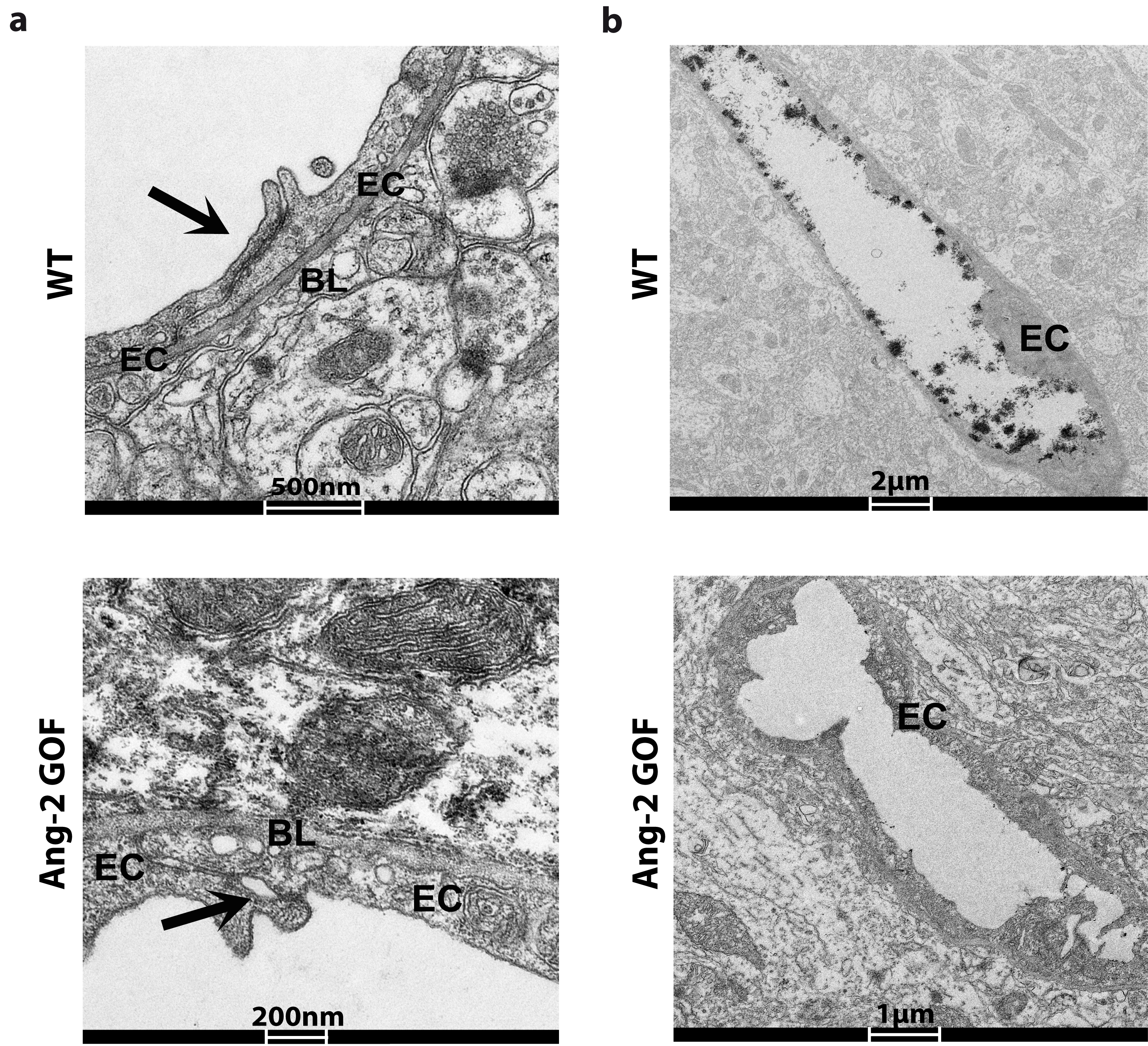

Supplement: Supplementary file 2 — Supplementary material 2 (TIFF 25692 kb) [file 401_2016_1551_MOESM2_ESM.tif]

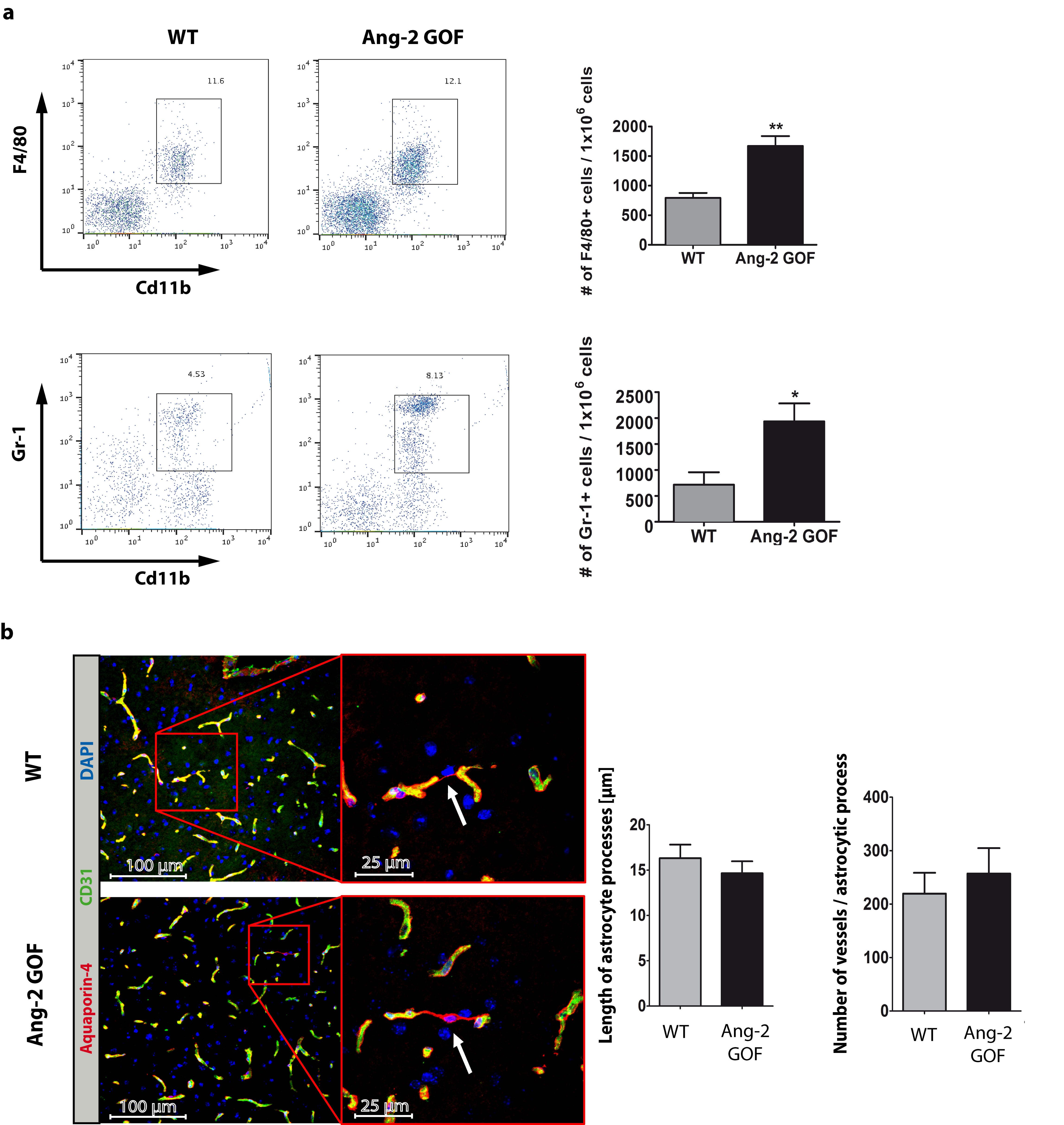

Supplement: Supplementary file 3 — Supplementary material 3 (TIFF 65399 kb) [file 401_2016_1551_MOESM3_ESM.tif]

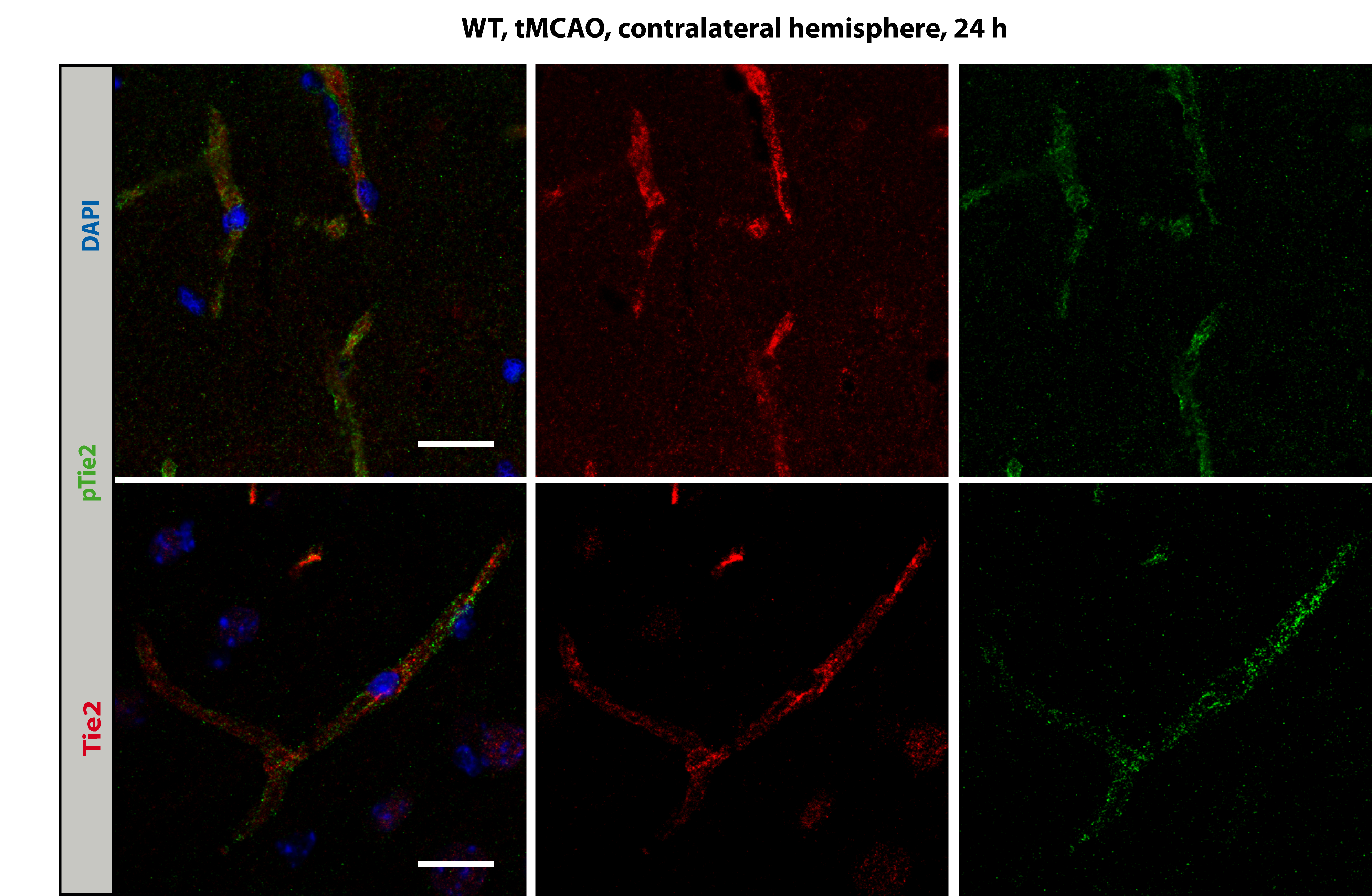

Supplement: Supplementary file 5 — Supplementary material 5 (TIFF 30487 kb) [file 401_2016_1551_MOESM5_ESM.tif]
