# Supplementary material for: Angiopoietin-2-induced blood–brain barrier compromise and increased stroke size are rescued by VE-PTP-dependent restoration of Tie2 signaling
Source: Acta Neuropathol. 2016 Mar 1;131:753–73. doi: 10.1007/s00401-016-1551-3 (PMC4835530; doi:10.1007/s00401-016-1551-3)
Supplement: Supplementary file 6 — Supplementary material 6 (DOCX 35 kb) [file 401_2016_1551_MOESM6_ESM.docx]

**Supplementary Figure legends**

**Supplementary Fig. 1:** **a)** A representative image of the capacitance (Ccl) of MBMECs of Ang-2 GOF versus WT mice. **b)** 3 kD TXR tracer clearance at 4 min vs 2 h. Before perfusion with PBS the blood was taken from the heart followed by serum fluorescence analysis (RFUs - raw fluorescence units) in a plate reader (n=5, 2-tailed unpaired t-test). **c)** Tracer permeability in kidneys showing no difference between WT and GOF mice thus serving as a control for leaky endothelium (n=7) **d)** qPCR analysis of MBMV (2 brains / preparation) showed a dramatic increase of hAng-2 in endothelial cells whereas Tie2 expression remained at the same level as in the WT samples (n=3; 2-tailed unpaired t-test). **e)** Serum ELISA analysis of mouse Ang-1 indicates no compensatory increase in GOF mice (n=3, 2-tailed unpaired t-test). **f)** IHC analysis of brain Ang-1 expression confirms ELISA data with no change in vascular Ang-1 expression between WT and GOF mice with CD31 serving as a vessel marker (Scale bars 25 µm).

**Supplementary Fig. 2:** Ultrastructural analysis revealed modified vessels in Ang-2 GOF mice. Representative images from cortex region are shown in the pictures. AEF=astrocytic endfeet; BL=basal-lamina; EC=endothelial cell; PC=pericyte. **a)** GOF mice exhibited gaps (arrows) between endothelial cells, which are normally connected in WT mice (arrows). **b)** GOF mice also showed decreased and disrupted glycocalyx formation with lanthanum nitrate staining compared to WT mice (n=3).

**Supplementary Fig. 3: a)** Flow cytometry analysis evidenced increased numbers of myeloid cells (macrophages and myeloid-derived suppressor cells (MDSCs)) in Ang-2 GOF mouse brains compared to WT. Macrophages were identified as CD45^hi^CD11b^+^Gr-1^-^F4/80^+^ cells, MDSCs as CD45^hi^CD11b^+^Gr-1^+^ (n=3 each, unpaired t-test). **b)** Cryosections of WT and Ang-2 GOF brains were stained for aquaporin-4 as an astrocyte endfeet marker (red) and CD31 (green) as a vessel marker. The number and length of astrocytic endfeet processes normalized to the number of vessels were analyzed (n=6-7 mice, 2-tailed unpaired t-test). No differences in these parameters were detectable between WT and Ang-2 GOF mice.

**Supplementary Fig. 4**: **a)** qPCR analysis of WT and Ang-2 GOF revealed no differences in mRNA levels in Meca 32 and Mfsd2a. Values were normalized to CD31 (n=3, 2-tailed unpaired t-test). **b)** MBMECs were treated with hAng-2 alone or in combination with AKB9785. The analysis showed decreased capacitance (Ccl) values in double-treated MBMECs (n=3, 2-tailed unpaired t-test) indicating tighter BBB in vitro. **c)** MBMECs showed a trend in increase in permeability to 67 kD BSA-Alexa 647 upon Ang-2 that was rescued by AKB-9785. No such effect was present upon treatment with filipin (10 nM). **d)** Western Blot analysis revealed no upregulation of pAkt in pericytes nor astrocytes after AKB-9785 treatment compared to hAng-2 alone. **e)** 3 kD TMR-dextran tracer permeability 3 h post stroke. In the cortex and sub-white matter (SWM) area increased extravasation of TMR was detectable in Ang-2 GOF mice (n=5, scale bars 10 µm).

**Supplementary Fig. 5:** Tie2/pTie expression in adult mouse brain. Representative images of the contralateral hemisphere of WT mice, 24h post-tMCAO. pTie2 (green), Tie2 (red) (n=4, scale bars 10 µm).

**Supplementary Table 1:**

| **Animal** | **Pericyte coverage [%]** | **Pericyte degeneration [%]** |
| --- | --- | --- |
| WT_1 | 100 | 0 |
| WT_2 | 100 | 0 |
| WT_3 | 98 | 0 |
| WT_4 | 98 | 0 |
| WT_5 | 99 | 0 |
| Ang-2 GOF_1 | 97 | 3 |
| Ang-2 GOF_2 | 86 | 2 |
| Ang-2 GOF_3 | 89 | 1 |
| Ang-2 GOF_4 | 97 | 3 |
| Ang-2 GOF_5 | 97 | 5 |

**Supplementary Table 2:**

| **Animal** | **Vessels with gaps within EC junctions [%]** |
| --- | --- |
| WT_1 | 0 |
| WT_2 | 0 |
| WT_3 | 0 |
| WT_4 | 0 |
| WT_5 | 0 |
| Ang-2 GOF_1 | 1 |
| Ang-2 GOF_2 | 2 |
| Ang-2 GOF_3 | 6 |
| Ang-2 GOF_4 | 3 |
| Ang-2 GOF_5 | 5 |

**Supplementary Table 3:**

| **Animal** | **Vessels containing HRP+ vesicles** | **Detected HRP+ vesicles** | **Vessels with HRP+**  **EC junctions** |
| --- | --- | --- | --- |
| WT_1 | 5 | 5 | 0 |
| WT_2 | 4 | 4 | 0 |
| WT_3 | 5 | 5 | 0 |
| Ang-2 GOF_1 | 27 | 93 | 5 |
| Ang-2 GOF_2 | 24 | 92 | 4 |
| Ang-2 GOF_3 | 27 | 149 | 8 |

**Supplementary Table 4:**

| **Type of stroke** | **Age** | **Gender** | **Diagnosis** | **Ang-2 expression [pg/ml]** | **Days after stroke** | **Infarct sizes** |
| --- | --- | --- | --- | --- | --- | --- |
| Control (healthy volunteer) | 25-40 | male | - | 2002 | - | - |
| Control (healthy volunteer) | 25-40 | female | - | 1374.48 | - | - |
| Control (healthy volunteer) | 25-40 | n.a. | - | 2120.42 | - | - |
| Control (healthy volunteer) | 25-40 | n.a. | - | 2089.08 | - | - |
| Control (healthy volunteer) | 28 | male | - | 2455.9 | - | - |
| Control (healthy volunteer) | n.a. | male | - | 2560.1 | - | - |
| Control (healthy volunteer) | 41 | male | - | 4829.8 | - | - |
| Control (healthy volunteer) | 27 | male | - | 2463 | - | - |
| Control (healthy volunteer) | 26 | female | - | 1668.3 | - | - |
| Control (healthy volunteer) | 36 | male | - | 2061.6 | - | - |
| Control (healthy volunteer) | 32 | female | - | 2741.7 | - | - |
| Control (healthy volunteer) | 45 | male | - | 2787 | - | - |
| Control (healthy volunteer) | 25 | female | - | 2028.4 | - | - |
| Control (healthy volunteer) | 34 | male | - | 2702.5 | - | - |
| Control (healthy volunteer) | 27 | female | - | 1705.6 | - | - |
| Control (healthy volunteer) | 30 | female | - | 518.4 | - | - |
| Lacunar stroke | 76 | female | small lacunar infarct | 3807.8 | 11 | 1 |
| Lacunar stroke | 66 | male | lacunar pontine infarct | 1355.82 | 6 | 1 |
| Lacunar stroke | 79 | male | lacunar infarct | 1759.08 | 2 | 1 |
| Lacunar stroke | 48 | male | lacunar infarct | 2081.22 | 2 | 1 |
| Territorial infarction | 83 | female | partial territorial infarct (middle cerebral artery); atrial fibrillation | 6080.72 | 15 | 4 |
| Territorial infarction | 65 | female | partial territorial infarct (middle cerebral artery); atrial fibrillation | 4620.7 | 5 | 4 |
| Territorial infarction | 62 | male | thalamic infarct, endocarditis | 3181.14 | 5 | 2 |
| Territorial infarction | 77 | female | cerebellar infarcts | 2881.54 | 1 | 2 |
| Territorial infarction | 81 | n.a. | partial territorial infarct (middle cerebral artery); atrial fibrillation | 7095.84 | 6 | 4 |
| Territorial infarction | 77 | female | partial territorial infarct (anterior and middle cerebral artery); thromboembolic | 2719.26 | 12 | 4 |
| Territorial infarction | 88 | female | territorial infarct (posterior cerebral artery); atrial fibrillation | 3464.78 | 3 | 4 |
| Territorial infarction | 79 | n.a. | small territorial infarct (middle cerebral artery); atrial fibrillation | 1893.82 | 9 | 3 |
| Territorial infarction | 89 | female | small brain stem infarct | 2384.12 | 2 | 3 |
| Territorial infarction | 72 | male | small territorial infarct (middle cerebral artery); severe risk factors | 8572.8 | 2 | 3 |
| Territorial infarction | 88 | n.a. | partial territorial infarct (middle cerebral artery) | 4536.98 | 12 | 4 |
| Territorial infarction | 68 | n.a. | large territorial infarct (middle cerebral artery) | 1762,12 | 7 | 5 |
| Territorial infarction | 70 | n.a. | small thromboembolic infarcts | 2072.26 | 8 | 1 |
| Territorial infarction | 72 | male | large territorial infarct (middle cerebral artery) | 3877.4 | 12 | 5 |
| Territorial infarction | 87 | female | large territorial infarct (middle cerebral artery) | 4373.94 | 9 | 5 |

**(n.a. not available)**

**Supplementary Table 5:**

| **Infarct grade** | **Age** | **Gender** | **Cause of death and /or main diseases in past-medical history** | **Localization** |
| --- | --- | --- | --- | --- |
| acute infarct (stage I) | 58 | female | liver cirrhosis | bilateral occipital |
| acute infarct (stage I) | 50 | female | gastric cancer, multiple organ failure, hemolysis | right basal ganglia |
| acute infarct (stage I) | 61 | male | lung carcinoma | multiple infarcts |
| acute infarct (stage I) | 62 | male | pneumonia, sepsis | left parieto-occipital |
| acute infarct (stage I) | 80 | male | electromechanical dissociation (EMD) | territorial infarct (right middle cerebral artery) |
| acute infarct (stage I) | 74 | female | multiple organ failure, sepsis | right occipital |
| acute to subacute infarct  (stage I – II) | 22 | female | acute myeloid leukemia, respiratory failure, liver failure, spleen infarct | left hemispheric |
| acute to subacute infarct  (stage I – II) | 69 | male | heart aneurysm, multiple organ failure | left occipital |
| acute to subacute infarct  (stage I – II) | 69 | female | chronic obstructive pulmonary disease (COPD), pneumonia, sepsis | right basal ganglia |
| subacute infarct (stage II) | 53 | male | extrapontine myelinolysis, alcohol abuse | left temporal |
| subacute infarct (stage II) | 74 | male | multiple organ failure, sepsis | left cerebellar |
| subacute infarct (stage II) | 74 | female | heart failure, sepsis | left temporo-basal |
| subacute infarct (stage II) | 71 | male | multiple organ failure | right parietal |
| subacute infarct (stage II) | 74 | female | lung carcinoma, chronic obstructive pulmonary disease (COPD) | left occipital |
| subacute infarct (stage II) | 87 | female | acute renal failure, pneumonia, ischemic cardiomyopathy, heart failure | right basal ganglia |
| subacute infarct (stage II) | 70 | male | sepsis, shock | left cerebellar |
| subacute infarct (stage II) | 79 | male | dementia, dehydration | right occipital |
| resolved infarct (stage III) | 68 | male | prostate cancer, carcinoma metastases | right basal ganglia |
| resolved infarct (stage III) | 81 | male | myocardial infarct | right basal ganglia |
| resolved infarct (stage III) | 76 | male | pneumonia, sepsis | left cerebellar |
| resolved infarct (stage III) | 67 | male | lung carcinoma, renal failure, myocardial ischemia | left basal ganglia |
| resolved infarct (stage III) | 71 | male | myocardial ischemia, liver cirrhosis, oral cancer | left basal ganglia |
| resolved (stage III) | 70 | male | heart failure, heart infarct, dementia, alcohol abuse, pneumonia | left basal ganglia |
| resolved infarct (stage III) | 70 | male | liver cirrhosis, liver cancer | left posterior |
| resolved infarct (stage III) | 75 | male | pneumonia, sepsis, chronic obstructive pulmonary disease (COPD) | territorial infarct (left middle cerebral artery) |
| resolved infarct (stage III) | 73 | male | heart failure | left cerebellar |
| resolved infarct (stage III) | 48 | female | sepsis, liver cirrhosis | left frontal |
| resolved infarct (stage III) | 69 | male | lung cancer, brain metastases | right temporal |

**Supplementary Table 6:**

| **Figure Nr.** | **number of animals** | **gender** | **age** | **body weight** | **blinded** | **comments** |
| --- | --- | --- | --- | --- | --- | --- |
| 1a | n=3; 4-6 mice per preparation | male and female | 8-12 weeks | 25-30g | no |  |
| 1b | n=3; 4-6 mice per preparation | male and female | 8-12 weeks | 25-30g | no |  |
| 1c | n=3; 4-6 mice per preparation | male and female | 8-12 weeks | 25-30g | no |  |
| 2a | 0.45 kD LY: WT n=6, GOF n=7  3 kD dextran: WT n=12, GOF n=16 | male and female | 8-12 weeks | 25-30g | no |  |
| 2b | n=6 | male and female | 8-12 weeks | 25-30g | no |  |
| 3a | n=8 | male and female | 8-12 weeks | 25-30g | no |  |
| 3b | n=3 | male and female | 8-12 weeks | 25-30g | no |  |
| 4b | n=3 | male and female | 8-12 weeks | 25-30g | no |  |
| 4c | n=5 | male and female | 8-12 weeks | 25-30g | no |  |
| 4d | n=5 | male and female | 8-12 weeks | 25-30g | no |  |
| 4e | n=5 | male and female | 8-12 weeks | 25-30g | no |  |
| 5a | n=3 | male and female | 8-12 weeks | 25-30g | no |  |
| 5b | n=3 | male and female | 8-12 weeks | 25-30g | no |  |
| 7a | 24 h n=4; 72 h WT n=10, GOF n=7; 7d n=5 | male and female | 8-12 weeks | 25-30g | no | 24 h_WT: 2 out of 6 mice had no stroke or died  24 h_GOF: 2 out of 6 mice had no stroke or died  72 h_WT: all 10 mice survived  72 h_GOF: 1 out of 8 mice had no stroke or died  7 d_WT: 1 out of 6 mice had no stroke or died  7d_GOF: 1 out of 6 mice had no stroke or died |
| 7b | WT n=4; GOF n=6 | male and female | 8-12 weeks | 25-30g | no |  |
| 8a | MBMECs n=3 | - | - | - | no |  |
| 8b | bEnd 5 cells n=3 | - | - | - | no |  |
| 8c | n=3 | - | - | - | no |  |
| 8d | n=3 | - | - | - | no |  |
| 9a | WT n=13; AKB n=14 | male | 8 weeks | 25-30g | yes |  |
| 9b | 24 h: Control n=8; AKB n=9  72 h: Control n=7; AKB n=9 | male | 8 weeks | 25-30g | yes | initially: 12 mice for control; 12 mice for AKB9785 teratment;  24 h: Control-3 animals died, 1 animal had no stroke; AKB-3 animals died  72 h: Control-1 animal died |
| 9c | Control n=6; AKB n=8 | male | 8 weeks | 25-30g | no |  |
| 9d | n=7 | male | 8 weeks | 25-30g | no |  |
| 9e | n=5 | male | 8 weeks | 25-30g | no |  |
| Suppl. 1a | n=6 | male and female | 8-12 weeks | 25-30g | no |  |
| Suppl. 1b | 2 brains per preparation; n=3 | male and female | 8-12 weeks | 25-30g | no |  |
| Suppl. 1c | n=3 | male and female | 8-12 weeks | 25-30g | no |  |
| Suppl. 2 | n=6 | male and female | 8-12 weeks | 25-30g | no |  |
| Suppl. 4a | 2 brains per preparation; n=3 | male and female | 8-12 weeks | 25-30g | no |  |
| Suppl. 4b | n=3 | male and female | 8-12 weeks | 25-30g | no |  |
| Suppl. 4c | n=3 | mixed | 8-12 weeks | 25-30g | no |  |
| Suppl. 4e | n=5 | Male | 8-12 weeks | 25-30g | no | initially: 5 WT, 6 GOF  1 WT died during surgery |
